# Formulation and Optimization of Artemether-Loaded Nanoemulsions by Applying QbD

**DOI:** 10.3390/ph19081264

**Published:** 2026-08-11

**Authors:** Yahya Alhamhoom, Umme Hani, Nagashubha Bobbarjang, Md Abdur Rashid, Bhargav Eranti, Battula Venkatesh, Fahad AlQahtani, Helal A. Helal, Mahesh Vaggu, Maccha Kiran Sai

**Affiliations:** 1 Department of Pharmaceutics, College of Pharmacy, King Khalid University, Abha 62223, Saudi Arabia; ysalhamhoom@kku.edu.sa (Y.A.); uahmed@kku.edu.sa (U.H.); mdrashid@kku.edu.sa (M.A.R.); 2 Department of Pharmaceutics, Raghavendra Institute of Pharmaceutical Education and Research Campus, Ananthapuramu 515721, Andhra Pradesh, India; bhargaveranti@yahoo.com (B.E.); bvenkateshlakshmi101@gmail.com (B.V.); vaggu.mahesh2020@gmail.com (M.V.); kiransaimks15502@gmail.com (M.K.S.); 3 Department of Pharmaceutical Sciences, College of Pharmacy, Umm Al-Qura University, Makkah 21955, Saudi Arabia; fjqahtani@uqu.edu.sa (F.A.); hahelal@uqu.edu.sa (H.A.H.)

**Keywords:** Artemether, Vippa oil, Sunflower oil, bioavailability, Box–Behnken design, tween 80, span 80, nanoemulsion

## Abstract

**Background**: Nanoemulsions are colloidal drug delivery systems consisting of an oil phase dispersed in water and stabilized by surfactants, producing droplets in the nanometer range. By virtue of their small droplet size and large interfacial area, they enhance drug dissolution, intestinal absorption, and site-specific delivery while offering controlled, prolonged release and a reduced risk of systemic side effects. Artemether (ART), an antimalarial agent, suffers from poor aqueous solubility and limited oral bioavailability, which restricts its therapeutic efficacy. **Objective**: The present study aimed to develop and optimize an Artemether-loaded nanoemulsion to improve the drug’s dissolution rate and oral bioavailability. **Methods**: The nanoemulsion was formulated using a combination of Sunflower oil and Vippa oil as the oil phase, with Tween 80 and Span 80 as the surfactant system, and was prepared by an ultrasonication technique. A three-factor, three-level Box–Behnken Design (BBD) was employed to systematically optimize the formulation composition and processing parameters. The formulations were evaluated for droplet size, polydispersity index (PDI), zeta potential, drug content, entrapment efficiency, pH, viscosity, refractive index, electrical conductivity, and cumulative in vitro drug release. **Results**: The optimized nanoemulsion exhibited a droplet size of 139.6 ± 1.3 nm, a PDI of 0.256 ± 0.03 indicating a narrow and uniform size distribution, and a zeta potential of −30.08 ± 1.1 mV reflecting good physical stability. The formulation demonstrated a high entrapment efficiency of 95.42 ± 1.18%, confirming efficient drug loading within the lipid core. Additional physicochemical evaluation revealed a pH of 6.4 ± 0.2, a low viscosity of 2.84 ± 0.15 cP, a refractive index of 1.338 ± 0.002, and a conductivity of 215 ± 12 µS/cm, collectively confirming the formation of a physiologically compatible, isotropic oil-in-water nanoemulsion. The formulation achieved 97.90 ± 0.97% cumulative in vitro drug release over a 12 h period, demonstrating a sustained release profile. **Conclusions**: The optimized Artemether-loaded nanoemulsion, developed using a Box–Behnken Design, significantly enhanced the drug’s dissolution and exhibited favorable physicochemical characteristics, high entrapment efficiency, and controlled release behavior. These findings suggest that nanoemulsion is a promising carrier system warranting further in vivo evaluation to confirm its potential for improving the oral bioavailability of Artemether.

## 1. Introduction

Nanoemulsions are colloidal systems formed by the homogeneous blending of two mutually insoluble liquids, stabilized by surfactants and co-surfactants that preserve a thermodynamically consistent single phase [1]. Their potential as platforms for drug delivery has attracted considerable scientific investigation [2]. Among the earliest formulations were oil-in-water (O/W) systems, defined by droplet sizes ranging between 50 and 1000 nm in diameter. Based on their internal architecture, nanoemulsions are broadly categorized into three structural types: O/W, in which oil globules are dispersed throughout an aqueous continuous phase; W/O, where water droplets are embedded within an oily matrix; and bi-continuous systems, in which both aqueous and lipid domains coexist in an interpenetrating network [3]. When both O/W and W/O configurations are simultaneously present within a single formulation, the resulting system is referred to as a multiple emulsion, which necessitates the concurrent use of both hydrophilic and lipophilic surfactants to achieve dual-phase stabilization [4]. From a therapeutic standpoint, nanoemulsions offer substantial benefits for the oral administration of poorly water-soluble compounds. They achieve this by enhancing the apparent aqueous solubility of such compounds and by providing a protective microenvironment that limits enzymatic degradation within the gastrointestinal tract. The incorporated surfactants and co-surfactants further contribute by modulating the permeability of intestinal membranes, facilitating more efficient drug absorption. Notably, nanoemulsion-based formulations have also demonstrated the capacity to minimize pharmacokinetic variability, yielding more consistent drug exposure profiles across diverse patient populations [4].

Among the various lipid-based nanocarrier platforms available for poorly water-soluble drugs, an oil-in-water (O/W) nanoemulsion was selected over alternative systems such as Self-Nanoemulsifying Drug Delivery Systems (SNEDDS), Solid Lipid Nanoparticles (SLNs), and Nanostructured Lipid Carriers (NLCs) for several formulation-specific reasons. SNEDDS typically require high surfactant concentrations (often 30–60% *w*/*w*) to achieve spontaneous self-emulsification upon dilution in gastrointestinal fluids, raising concerns regarding surfactant-induced mucosal irritation and dose-dependent gastrointestinal intolerance on chronic or repeated administration. In contrast, the nanoemulsion developed in this study achieved stable, sub-150 nm droplets using a comparatively modest surfactant load, reducing this risk while retaining the permeation-enhancing benefits associated with Tween 80 and Span 80.

SLNs and NLCs, meanwhile, rely on a solid or partially solid lipid matrix at body temperature, which constrains drug loading capacity for lipophilic actives such as ART, since a highly ordered crystalline lipid lattice provides limited accommodating space compared with a fully liquid oil core; this can also predispose SLN/NLC systems to drug expulsion during storage as the lipid matrix undergoes polymorphic transitions toward a more stable crystalline form. Garg et al. [5], for example, reported an ART-loaded SLN system with an entrapment efficiency of approximately 78%, notably lower than the 95.42 ± 1.18% entrapment efficiency achieved by the fully liquid dual-oil nanoemulsion core developed in the present study, consistent with the generally higher drug-loading capacity of liquid lipid systems for lipophilic actives. On this basis, an O/W nanoemulsion was considered the most suitable platform for maximizing ART solubilization and loading while minimizing excipient-related tolerability concerns.

Artemether (ART) is a lipid-soluble derivative of artemisinin, used alone or as a fixed-dose combination with lumefantrine as a first-line artemisinin-based combination therapy for uncomplicated falciparum malaria. Despite its potent and rapidly acting antimalarial action, the clinical performance of oral ART is constrained by its very low aqueous solubility, which results in poor and erratic dissolution-limited absorption. Even the fraction that is absorbed undergoes extensive first-pass metabolism, principally via the CYP3A4 isoenzyme, to its active metabolite dihydroartemisinin, and its short elimination half-life further contributes to variable, often subtherapeutic systemic drug exposure. Together, these solubility, permeability, and metabolic limitations mean that conventional oral dosage forms of ART, such as plain tablets or suspensions, frequently show incomplete and inter-individually variable absorption, underscoring the need for delivery strategies capable of improving the drug’s apparent solubility and dissolution rate at the intestinal level.

Several groups have investigated lipid-based nanocarriers to address these limitations. An early precedent for this approach in antimalarial therapy was established by Singh and Vingkar [2], who developed an oral lipid nanoemulsion of primaquine and demonstrated improved antimalarial activity and altered biodistribution relative to the plain drug. Building on this general strategy specifically for ART, Laxmi et al. [6] developed an ART nanoemulsion using coconut oil with Span 80 and Tween 80, prepared by ultrasonication, obtaining a droplet size of approximately 79 nm and a zeta potential of −15 mV; in Wistar rats, this formulation achieved an oral bioavailability approximately 2.6-fold higher than the plain drug. Working with the related artemisinin derivative arteether, Dwivedi et al. [7] prepared nanoemulsions by high-pressure homogenization and reported improved oral bioavailability, confirmed by LC–MS pharmacokinetic analysis, together with enhanced antimalarial efficacy in *Plasmodium yoelii nigeriensis*-infected mice. Beyond nanoemulsions, Garg et al. [5] formulated ART-loaded solid lipid nanoparticles (~362 nm, zeta potential −17.6 mV, ~78% entrapment efficiency) and, using DSC, demonstrated molecular dispersion of the drug within the lipid matrix together with improved intestinal permeability relative to the plain drug.

Beyond lipid-based systems, nanocarrier engineering more broadly has demonstrated considerable success in overcoming the biological barriers that limit the delivery of poorly bioavailable therapeutics. For instance, biomineralized inorganic nanocarriers have been employed to improve the in vivo stability and barrier penetration of macromolecular drugs [8], while biocompatible polyphenol-based nanoplatforms have been developed to enhance the intracellular delivery of protein therapeutics [9]. These examples illustrate the broader principle underlying the present work: that rationally engineered nanocarrier systems—whether lipid-, polymer-, or biomineral-based—can be tailored to the specific physicochemical and biological barriers relevant to a given therapeutic class, motivating the lipid nanoemulsion strategy adopted here for the small-molecule antimalarial Artemether.

Collectively, these studies confirm that lipid-based nanocarriers can improve the apparent solubility, dissolution, and/or intestinal permeability of ART and related artemisinin derivatives. However, the existing ART nanoemulsion literature has relied on conventional oils such as coconut oil and has not, to our knowledge, evaluated the non-conventional Vippa oil as an oil-phase component, nor combined systematic, statistically validated Quality by Design (QbD) optimization with ex vivo intestinal permeation testing under a unified experimental design. In the present work, Sunflower oil and Vippa oil were selected as the oil-phase components, and the ART nanoemulsion was optimized using a QbD approach employing a three-factor, three-level Box–Behnken statistical design, with formulation performance further assessed by ex vivo permeation across excised intestinal mucosa. This combination of a novel, non-conventional oil phase with a statistically driven QbD optimization framework and ex vivo permeation evaluation constitutes the specific novelty of the present study relative to prior ART/Artemether nanoemulsion work.

## 2. Results

### 2.1. HPLC Analysis of Artemether

**Figure 1:**
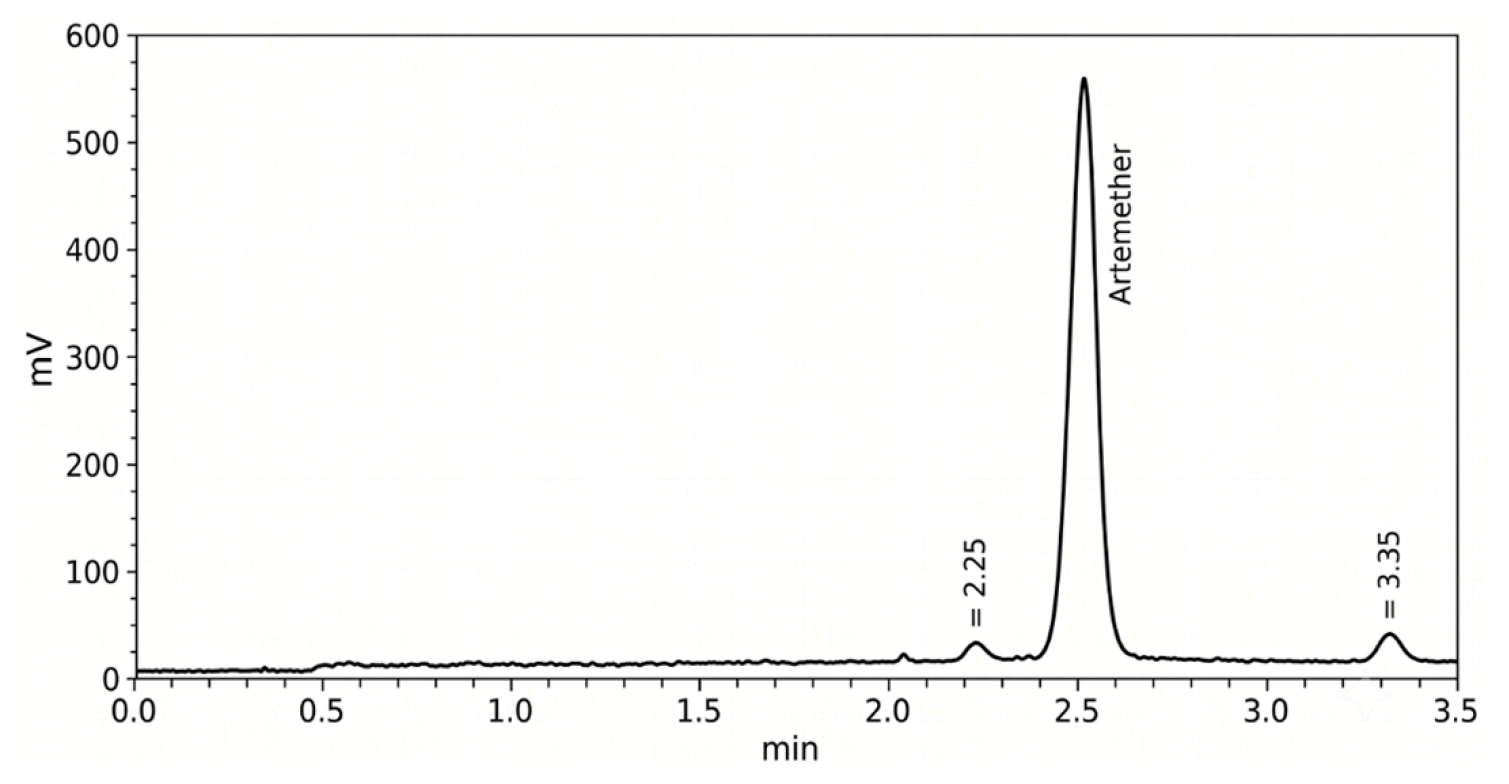
HPLC chromatogram of Artemether.

The chromatogram obtained from the analysis of the Artemether standard is shown in Figure 1. The chromatogram demonstrates a sharp, highly prominent, and symmetrical peak, confirming the successful identification and adequate resolution of Artemether under the established chromatographic conditions. Based on the data, the retention time for the main Artemether analyte is approximately 2.5 min, and its maximum signal intensity approaches 600 mV, indicating a strong detector response. The baseline appears exceptionally clean and stable, reflecting the presence of negligible detector noise and the use of well-equilibrated reagents.

Furthermore, the analytical method exhibits good sensitivity by resolving two smaller, distinct peaks that denote the presence of related compounds or minor impurities within the Artemether standard material. These secondary components were successfully eluted at retention times of roughly 2.25 min and 3.35 min, respectively. Despite their relatively low abundances compared to the principal peak, their clear resolution from the Artemether peak demonstrates the developed method’s selectivity for distinguishing related substances.

a. Sensitivity (LOD and LOQ)

The sensitivity of the UV-Visible spectrophotometric method was established through calculation of the LOD and LOQ. The LOD, defined as the lowest concentration of Artemether that can be reliably detected, was found to be 0.71 µg/mL, while the LOQ, defined as the lowest concentration that can be quantified with acceptable precision and accuracy, was found to be 2.15 µg/mL. Since the calculated LOQ (2.15 µg/mL) lies well below the lowest concentration of the linear calibration range (5 µg/mL), the method demonstrates adequate sensitivity for the accurate and reliable quantification of Artemether in both the nanoemulsion formulations and the dissolution/permeation media.

### 2.2. Solubility

The saturation solubility of Artemether in various aqueous and organic solvents, as well as selected lipidic excipients, was quantitatively determined to guide selection of the oil phase and surfactant system. The results are summarized in Table 1. Artemether exhibited negligible solubility in distilled water (0.02 ± 0.01 mg/mL), confirming its pronounced hydrophobic nature. The slightly improved solubility in phosphate buffer (pH 6.8, 0.15 ± 0.03 mg/mL) remains insufficient for standard oral delivery, reinforcing the necessity for a lipid-based nanocarrier. Among the tested oil phases, the non-conventional Vippa oil demonstrated a superior solubilization capacity for Artemether (68.40 ± 2.15 mg/mL) compared to Sunflower oil (45.25 ± 1.54 mg/mL), a result consistent with Artemether’s highly lipophilic (BCS Class II) character. This quantitative solubility differential provided the basis for selecting Vippa oil as the primary oil phase in the optimized dual-oil matrix, to maximize drug loading while minimizing the risk of precipitation. Span 80 similarly showed higher ART solubility (62.15 ± 1.90 mg/mL) than Tween 80 (55.30 ± 1.75 mg/mL), supporting the surfactant system selected for the formulation.

### 2.3. Screening and Selection of Excipients

The excipient selection based on solubility, the ranges of the independent variables for the Box–Behnken Design (Table 2) were established from preliminary phase-behavior trials conducted prior to formal optimization. Trial formulations spanning a wider composition range (aqueous phase: 60–95%; oil:surfactant ratio: 0.5–4.0%; sonication time: 2–15 min) were screened visually for clarity, phase separation, and creaming after 24 h at room temperature. Formulations with an aqueous phase below 75% showed visible turbidity/phase inversion, while those above 85% did not accommodate sufficient oil-phase volume for adequate drug loading. Oil:surfactant ratios below 1% resulted in incomplete emulsification of the oil phase, whereas ratios above 2% did not further improve clarity or stability. Similarly, sonication times below 5 min produced coarse, polydisperse emulsions, while times beyond 10 min offered no further reduction in droplet size. On this basis, the ranges of 75–85% (aqueous phase, A), 1–2% (oil:surfactant ratio, B), and 5–10 min (sonication time, C) were selected as the design space for the Box–Behnken optimization (Table 2). This preliminary range-finding step directly addresses the three Critical Process Parameters (CPPs) identified in the QbD risk assessment, ensuring that the subsequent Box–Behnken Design was conducted only after the practically formulable design space had been established, rather than over an arbitrarily wide or unscreened range.

### 2.4. FT-IR Studies

**Figure 2:**
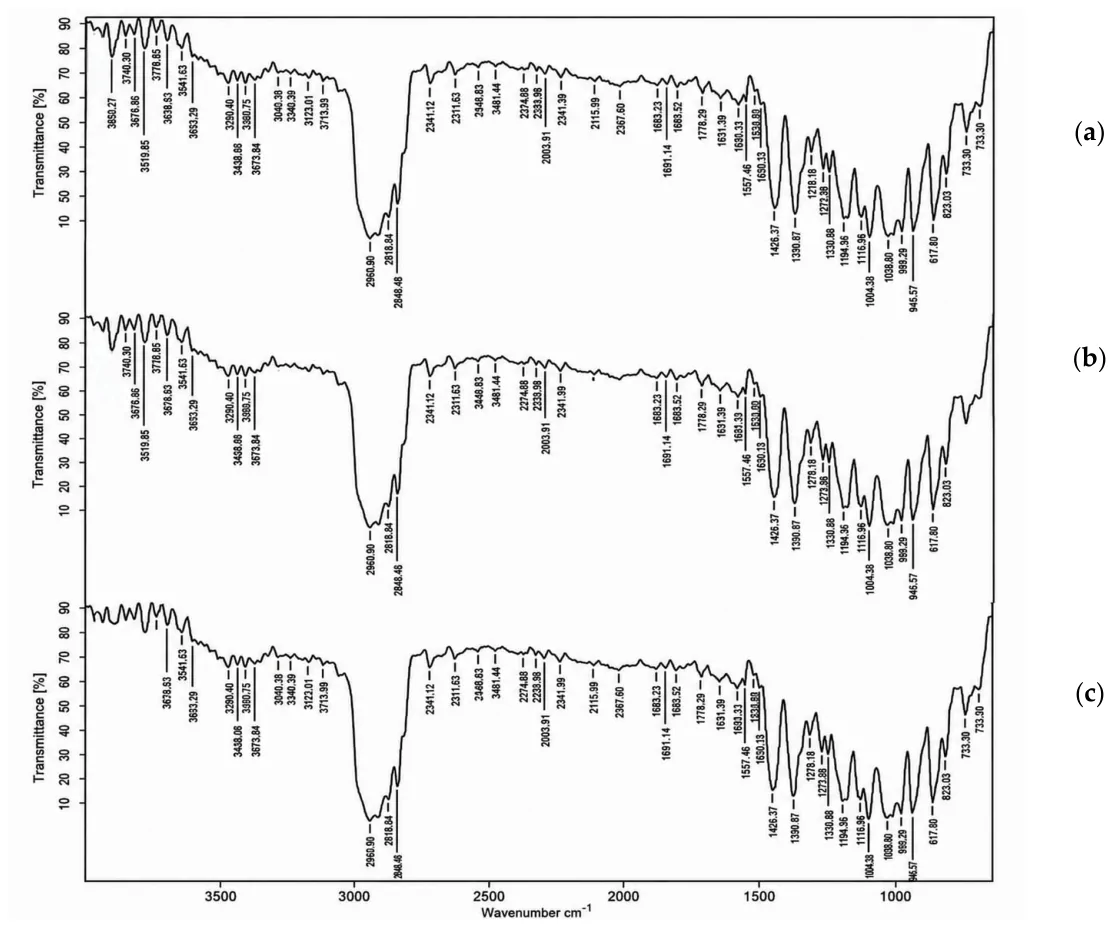
FT-IR spectrum of (**a**) pure drug (Artemether), (**b**) drug with excipients, and (**c**) formulation.

The FT-IR spectrum of pure Artemether exhibited characteristic absorption bands at 3172.9 cm^−1^ (aromatic C–H), 2946.8 cm^−1^ (aliphatic C–H stretching), 1715.3 cm^−1^ (C=O stretching), 1463.2 cm^−1^ (–CH_2_ bending), and 1104.6 cm^−1^ (C–O stretching), confirming the chemical identity of the drug (Figure 2, Table 2).

To assess drug–excipient compatibility, FT-IR spectra of the drug–excipient physical mixture and the optimized nanoemulsion were recorded under identical conditions and overlaid with that of the pure drug (Figure 2a). The three spectra were essentially superimposable: all principal absorption bands of Artemether were retained in both the physical mixture and the formulation at their original positions, with no measurable shift in the major functional-group vibrations, no disappearance of characteristic peaks, and no emergence of new bands. In particular, the diagnostic C=O stretching (1715.3 cm^−1^), –CH_2_ bending (1463.2 cm^−1^), and C–O stretching (1104.6 cm^−1^) bands remained unchanged across all three samples. The absence of any significant shift confirms that no chemical interaction occurred between Artemether and the selected excipients (Sunflower oil, Vippa oil, Tween 80, and Span 80), establishing their compatibility and the retention of drug integrity within the nanoemulsion (Figure 2b,c).

### 2.5. DSC Studies

**Figure 3:**
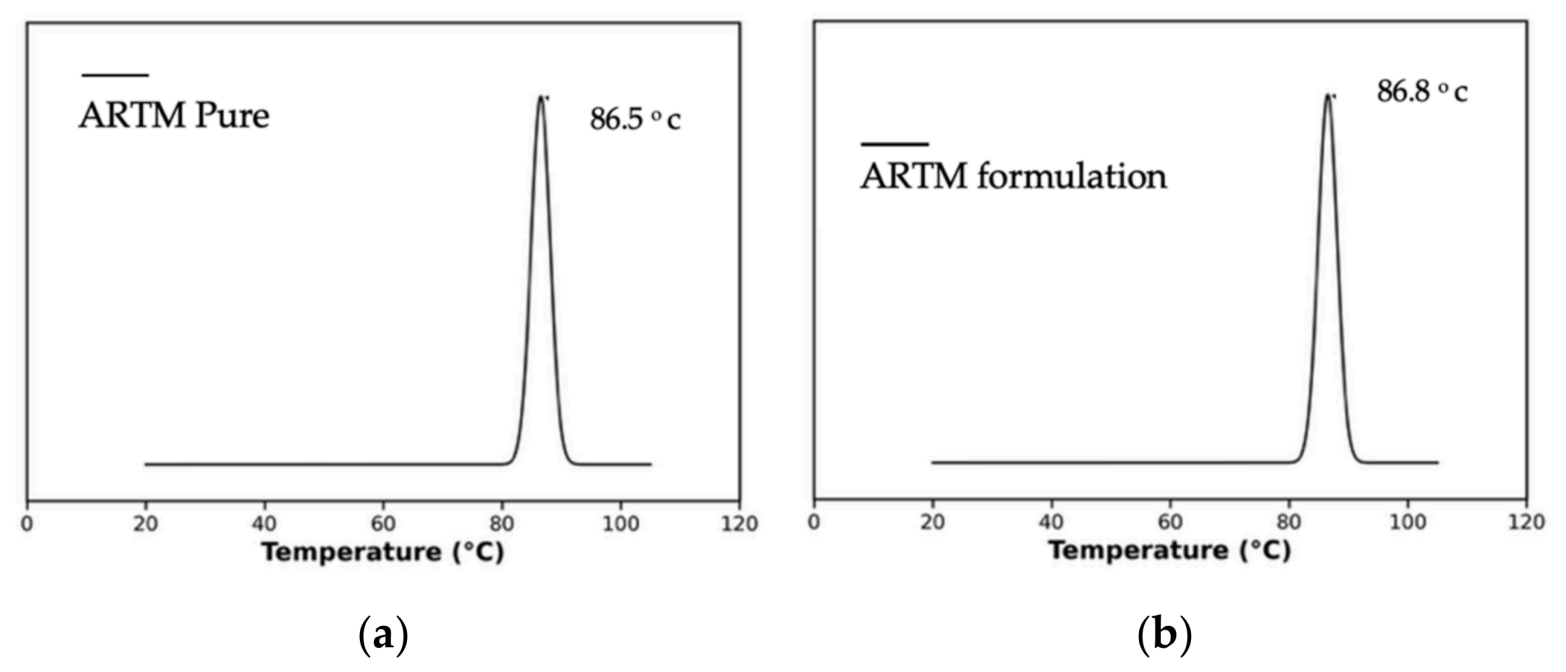
DSC thermogram of (**a**) pure drug (Artemether) and (**b**) formulation.

The DSC thermogram of pure Artemether and the Artemether-loaded nanoemulsion formulation showed an endothermic transition at 86.5 °C and 86.8 °C, respectively, corresponding to the reported melting point of ART. The enthalpy of fusion (ΔHm) was 78.5 J/g for pure Artemether, compared with only 8.4 J/g for the formulation, corresponding to a relative crystallinity of approximately 10.7% (i.e., an ~89.3% reduction in crystallinity) in the formulated product. Notably, the endothermic peak in the formulation was broader and of markedly reduced enthalpy compared with the sharp, well-defined melting endotherm of the pure crystalline drug, consistent with a substantial reduction in the proportion of ART present in an ordered crystalline state within the formulation. The thermogram is presented in Figure 3a,b.

### 2.6. Design of Experiments (DoE)

A Box–Behnken Design with three factors at three levels was employed. The independent variables and their levels are summarized in Table 2, and the 17 experimental runs with observed responses are presented in Table 3.

### 2.7. Particle Size and Polydispersity Index (F1–F17)

**Figure 4:**
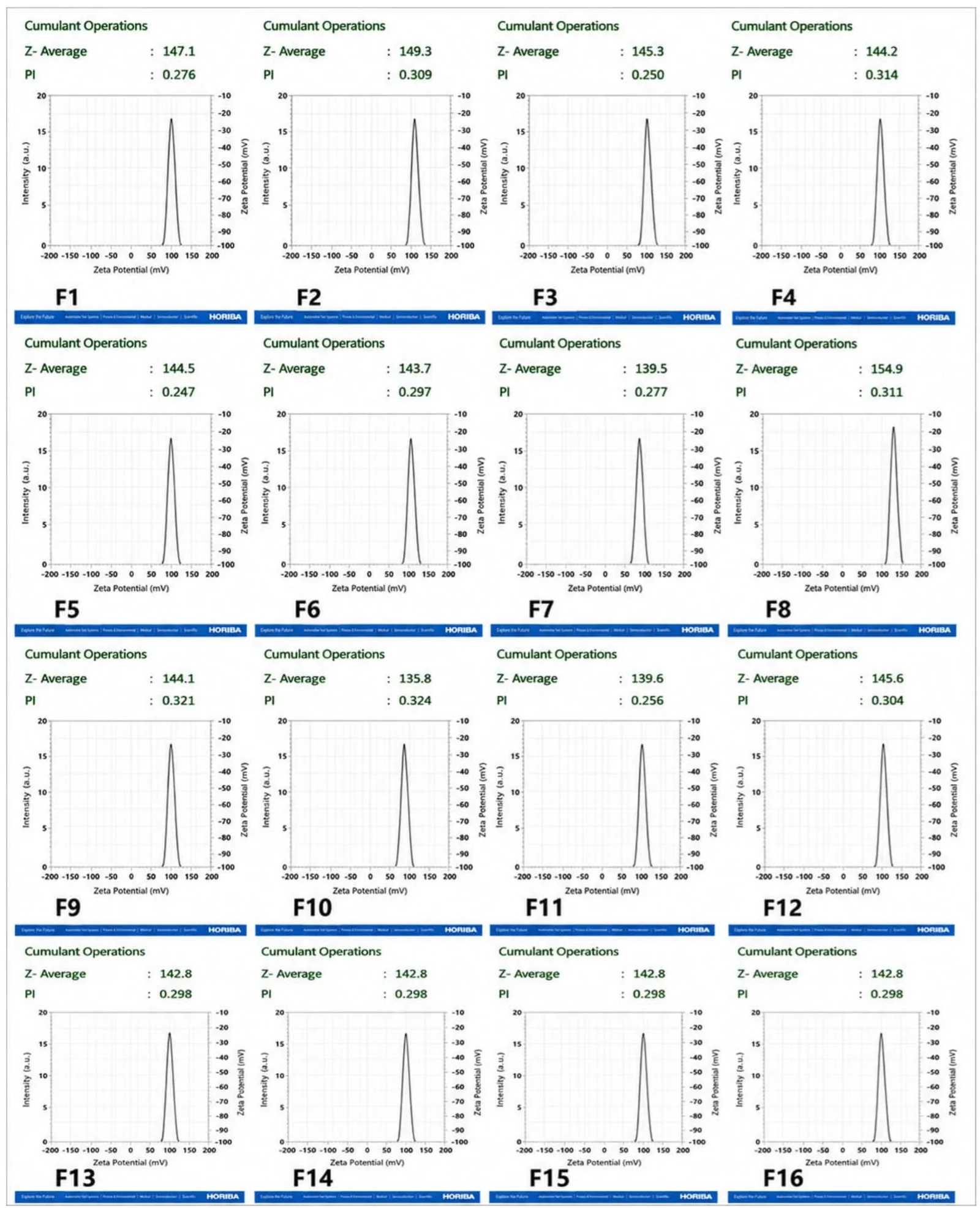
Particle size and polydispersity index of ART-NE formulations (F1–F17).

The measured particle size values ranged from 135.8 ± 1.1 nm to 154.9 ± 3.8 nm across all 17 formulations, and PDI values ranged from 0.247 ± 1.2 to 0.324 ± 0.8. All PDI values were below 1.0, confirming a narrow and homogeneous size distribution. Results are presented in Figure 4.

### 2.8. Zeta Potential

**Figure 5:**
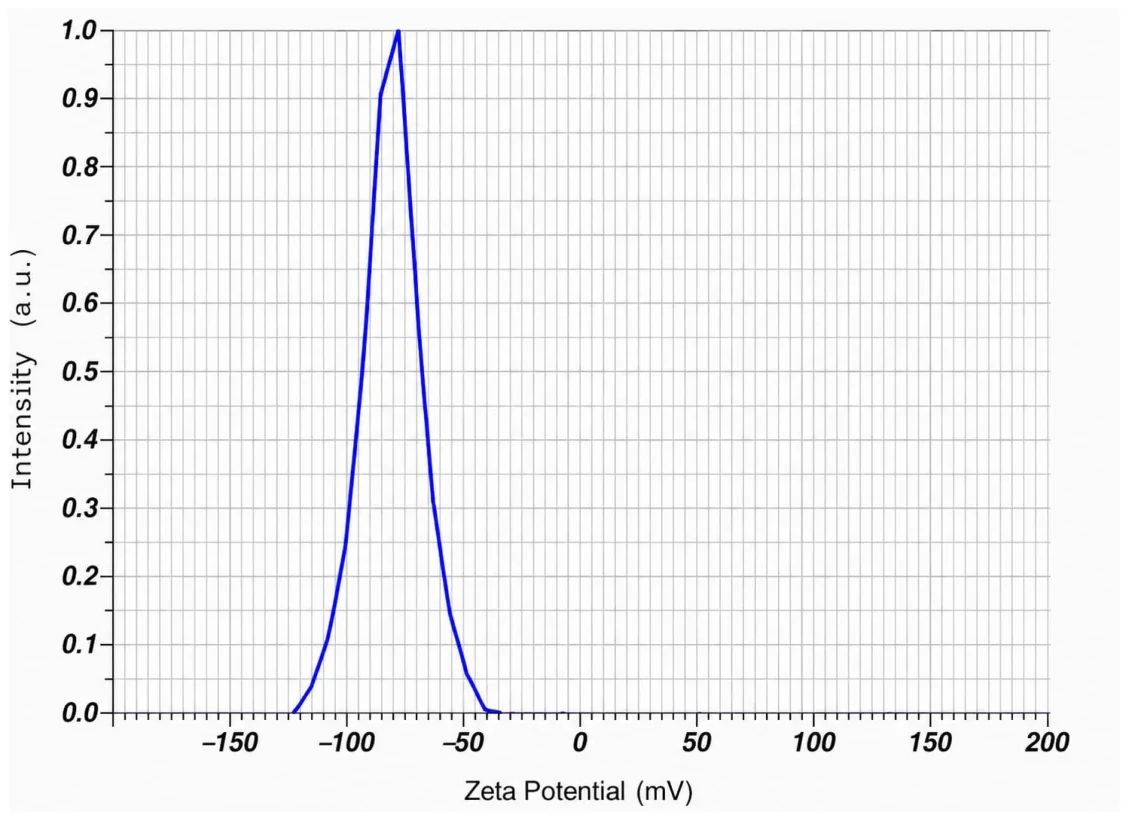
Zeta potential of optimized formulation.

The optimized ART-NE formulation exhibited a zeta potential of −30.08 mV, indicating adequate electrostatic repulsion between droplets and satisfactory long-term colloidal stability. The zeta potential profile is presented in Figure 5.

### 2.9. Additional Physicochemical Properties

The optimized Artemether-loaded nanoemulsion was evaluated for pH, viscosity, refractive index, and conductivity to ensure its suitability for oral administration and physical stability, as detailed in Table 4.

- pH: The pH of the formulation was found to be 6.4 ± 0.2, which falls within the acceptable physiological range for oral preparations, ensuring non-irritancy to the gastrointestinal mucosa.
- Viscosity: The low viscosity of 2.84 ± 0.15 cP is characteristic of an oil-in-water (O/W) nanoemulsion with a high aqueous phase volume (75%). This low viscosity is highly desirable as it facilitates easy pouring, swallowing, and rapid dispersion in gastrointestinal fluids.
- Refractive Index: The formulation exhibited a refractive index of 1.338 ± 0.002. This value is very close to that of pure water (1.333), confirming the isotropic, transparent-to-translucent nature of the nanoemulsion and verifying that the oil droplets are dispersed at a nanometric scale that does not significantly scatter light.
- Conductivity: The electrical conductivity was measured at 215 ± 12 µS/cm. This relatively high conductivity value conclusively confirms the formation of an oil-in-water (O/W) system, as water constitutes the continuous external phase allowing for electrical conductance, whereas a water-in-oil (W/O) system would exhibit conductivity approaching zero.

### 2.10. Drug Content

Drug content across all formulations ranged from 86.12% to 98.44%. Formulations incorporating Vippa oil and Sunflower oil as the oil phase consistently achieved higher drug content compared to other formulations. Results are presented in Table 5.

### 2.11. Entrapment Efficiency

The entrapment efficiency (EE%) of the optimized Artemether nanoemulsion was evaluated to determine the nanocarrier’s drug-loading capacity. The optimized formulation exhibited an exceptionally high entrapment efficiency of 95.42 ± 1.18% (n = 3). This result indicates that the vast majority of the incorporated Artemether was successfully encapsulated within the lipid matrix of the nanoemulsion, with only a negligible fraction remaining free in the continuous aqueous phase.

### 2.12. In Vitro Lipolysis (Biorelevant Digestion) Study

The distribution of ART across the aqueous micellar, oil, and precipitate phases following simulated intestinal digestion is summarized in Table 6. For the plain ART suspension, only 2.8 ± 0.5% of the drug dose was recovered in the aqueous micellar phase, with the overwhelming majority (97.2 ± 0.5%) found in the precipitate phase, indicating that free ART rapidly exceeds its solubility limit in the aqueous digestion medium and precipitates out rather than remaining available for absorption.

In marked contrast, the optimized ART-NE retained 78.4 ± 2.6% of the drug dose in the aqueous micellar phase, with 16.2 ± 1.4% remaining in the residual (partially digested) oil phase and only 5.4 ± 0.9% precipitating. This represents an approximately 28-fold increase in micellar solubilization relative to the plain suspension, and a corresponding near-total elimination of drug precipitation during digestion.

### 2.13. In Vitro Drug Release of ART-NEs

**Figure 6:**
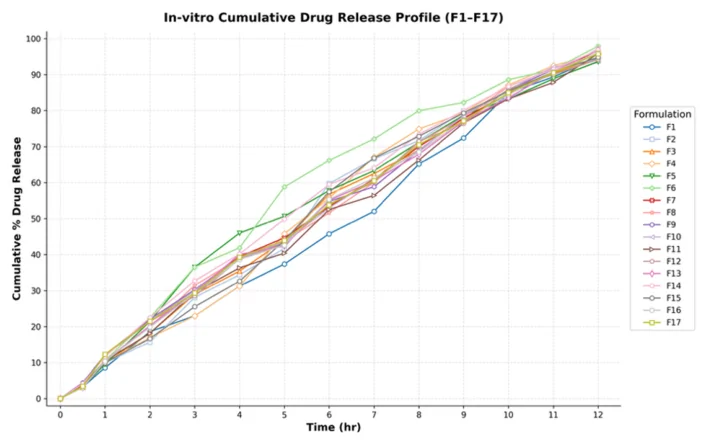
In vitro drug release profile of ART-NEs (F1–F17).

Cumulative in vitro drug release data for formulations F1–F17 over 12 h are presented in Table 7 and graphically in Figure 6. In vitro drug release profile of ART-NEs (F1–F17).

### 2.14. Ex Vivo Drug Release

**Figure 7:**
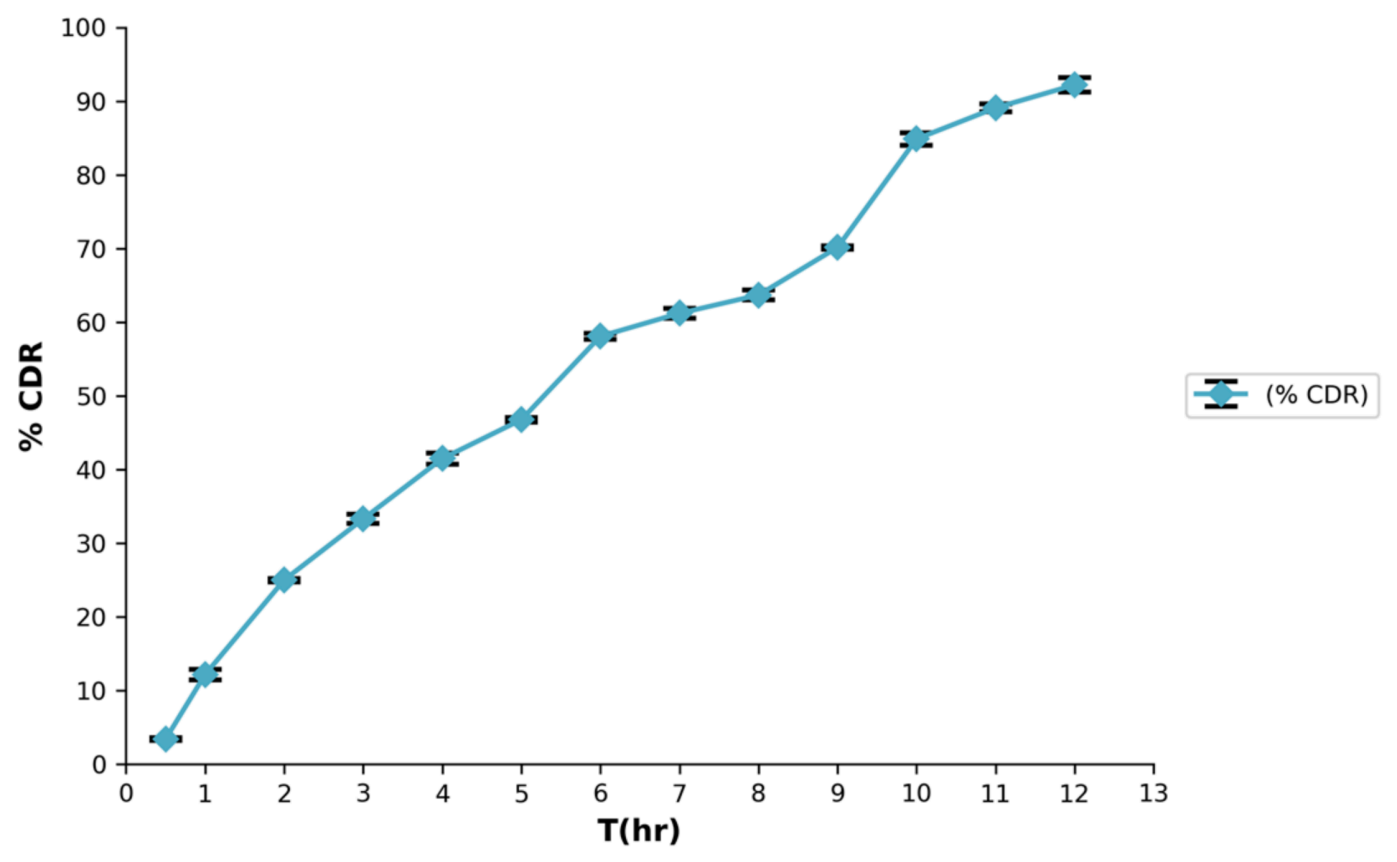
Ex vivo drug release profile of optimized formulation.

Cumulative ex vivo drug permeation data for the optimized formulation over 12 h are presented in Table 8 and Figure 7.

Drug release data were fitted to zero-order, first-order, Higuchi, and Korsmeyer–Peppas kinetic models. The R^2^ values and release exponent (n) for all formulations are summarized in Table 8. Zero-order, first-order, Higuchi, and Korsmeyer–Peppas kinetic plots of ART-NEs (F1–F17) are presented in Figure 6.

The permeation profiles of the plain ART suspension and the optimized ART-NE across excised goat intestinal mucosa are shown in Figure 7, and the corresponding permeation parameters, quantified by the validated RP-HPLC method, are summarized in Table 9. The plain ART suspension exhibited a steady-state flux (Jss) of 145.20 ± 6.14 µg/cm^2^/h and an apparent permeability coefficient (Papp) of (2.52 ± 0.11) × 10^−5^ cm/s, with a lag time of 1.85 ± 0.14 h and a cumulative permeation of only 24.35 ± 1.42% over 12 h, consistent with the drug’s inherently poor aqueous solubility and slow partitioning across the mucosal membrane.

In contrast, the optimized ART-NE achieved a markedly higher Jss of 586.45 ± 12.38 µg/cm^2^/h and a Papp of (1.02 ± 0.04) × 10^−4^ cm/s, corresponding to a 4.04 ± 0.18-fold enhancement in flux relative to the plain drug suspension. The lag time was also substantially and consistently reduced, from 1.85 ± 0.14 h to 0.42 ± 0.06 h, indicating a much faster and more reproducible onset of drug transport across the membrane. Cumulative permeation over 12 h reached 92.21 ± 0.97% for the nanoemulsion, compared with only 24.35 ± 1.42% for the plain drug suspension. The relatively small SD values across all parameters (n = 3), together with the specificity and resolution afforded by the HPLC method, indicate good inter-replicate reproducibility and confirm that the observed enhancement reflects a genuine formulation effect rather than an analytical artifact.

### 2.15. Drug Release Kinetics of ART-Nes

**Figure 8:**
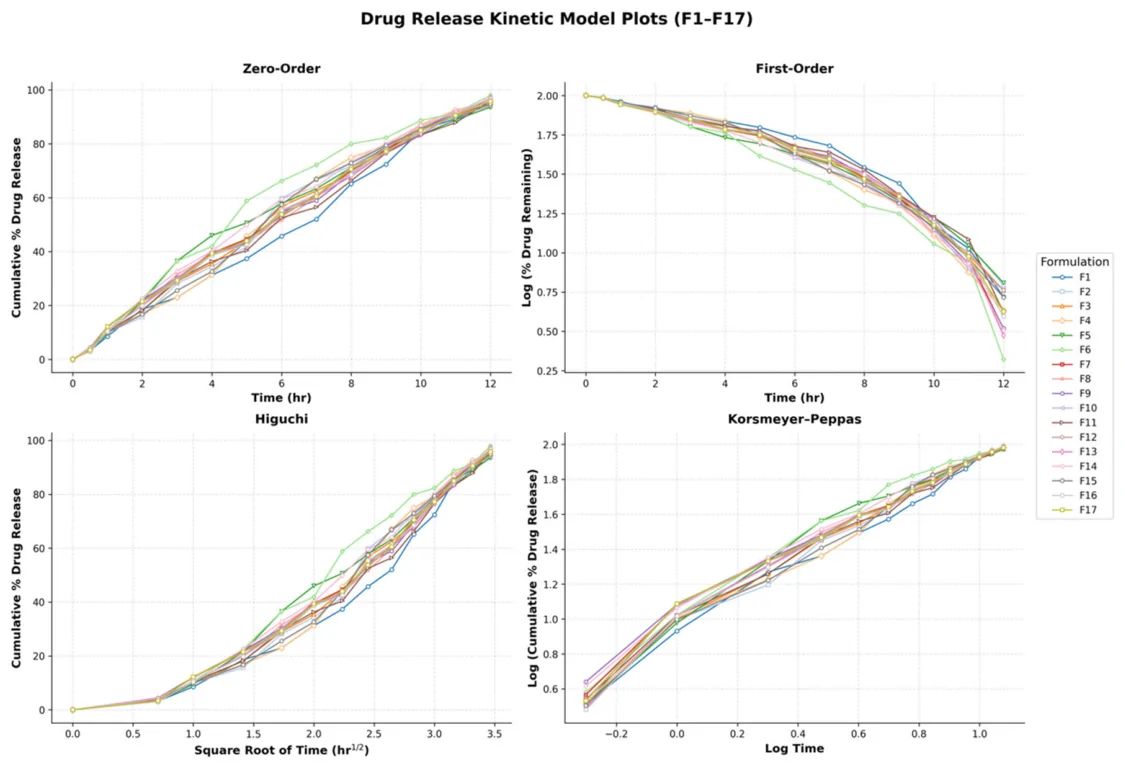
Zero-order, first-order, Higuchi, and Korsmeyer–Peppas kinetic plots of ART-NEs (F1–F17).

Drug release kinetic model plots (F1–F17): cumulative % drug release vs. time (zero-order), log % drug remaining vs. time (first-order), cumulative % drug release vs. square root of time (Higuchi), and log cumulative % drug release vs. log time (Korsmeyer–Peppas) in Figure 8.

The release behavior of all 17 formulations (F1–F17) was fitted to four kinetic models to identify the dominant drug transport mechanism, with the resulting plots shown in Figure 8. The zero-order plot shows a consistently linear increase in cumulative drug release across all formulations over the 12 h period, visually corroborating the high R^2^ values (0.963–0.995, Table 9) obtained for this model and confirming a constant, formulation-independent release rate. The first-order plot shows a comparatively less linear decline in log (% drug remaining), particularly in the later time points (10–12 h), consistent with the poorer fit of this model relative to zero-order kinetics across the design space.

The Higuchi plot displays a reasonably linear relationship between cumulative release and the square root of time for most formulations, supporting a diffusion-contributing component to drug release, while the Korsmeyer–Peppas plot shows a consistent, near-linear relationship across all 17 formulations with a shallow slope, corresponding to the release exponent (*n*) values of 0.13–0.39 obtained from the regression analysis (Table 10). As all n values fall below the 0.43 threshold for spherical matrices, this confirms Fickian diffusion as the predominant release mechanism across the entire formulation set, rather than being specific to the optimized batch alone.

Across all four models, formulation F17 (green) consistently shows a modestly faster release profile relative to the other 16 formulations, particularly evident in the zero-order and Higuchi plots, while F1–F16 cluster closely together, indicating that the Box–Behnken design space produced formulations with broadly comparable, reproducible release behavior with the exception of this single high-release outlier.

### 2.16. Diagnostic Plots for Particle Size (PS)

**Figure 9:**
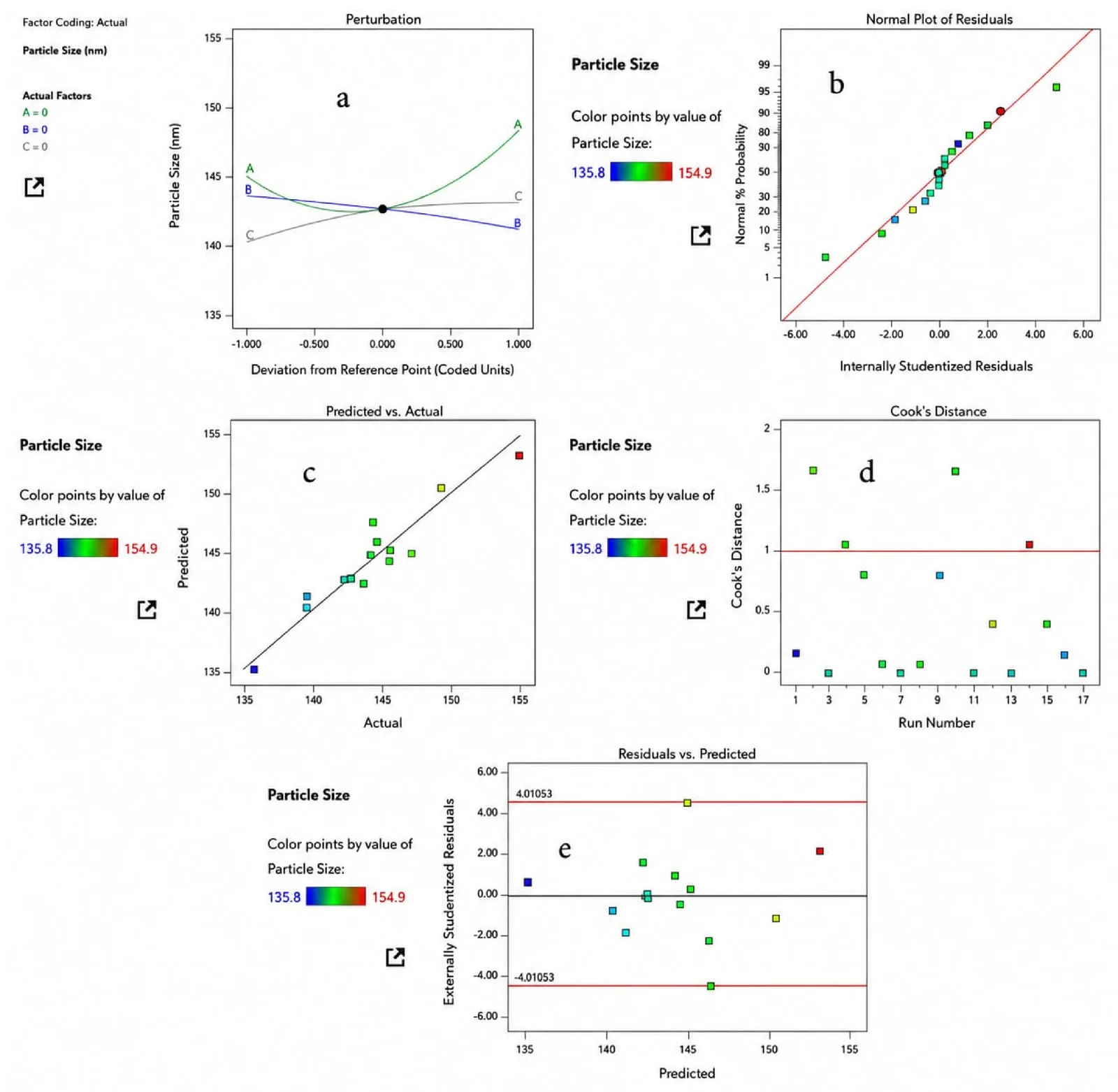
Diagnostic plots for the quadratic model of particle size: (**a**) perturbation chart, (**b**) normal probability plot of residuals, (**c**) predicted vs. actual plot, (**d**) Cook’s distance plot, and (**e**) residuals vs. predicted plot.

The adequacy of the fitted quadratic model for particle size was assessed using a set of diagnostic plots (Figure 9). The perturbation chart (Figure 9a) showed that the aqueous phase (factor A) produced the steepest and most curved response, indicating that it was the most influential variable on particle size, whereas the oil:surfactant ratio (B) and sonication time (C) produced comparatively flatter profiles. The normal probability plot of residuals (Figure 9b) showed the data points distributed closely along the straight line, confirming that the residuals were approximately normally distributed and that the assumption of normality was satisfied. The predicted vs. actual plot (Figure 9c) showed the points lying close to the line of equality, demonstrating good agreement between the model-predicted and experimentally observed particle size values. In the Cook’s distance plot (Figure 9d), most runs fell below the influence limit, although a few runs approached or slightly exceeded the threshold, indicating a small number of comparatively influential observations. Finally, the residuals vs. predicted plot (Figure 9e) showed the residuals scattered randomly around the central line and contained within the upper and lower control limits, with no systematic pattern, confirming constant variance of the residuals. Collectively, these plots indicate that the quadratic model adequately describes the experimental data for particle size.

### 2.17. Quadratic Regression ANOVA for Particle Size

The ANOVA results for the quadratic model are presented in Table 11, and the corresponding fit statistics in Table 12. The model F-value of 7.63 implies that the model is significant, with only a 0.69% probability that an F-value of this magnitude could arise due to noise. Among the individual terms, the aqueous phase (A, *p* = 0.0233), the interaction terms AC (*p* = 0.0040) and BC (*p* = 0.0074), and the quadratic term A^2^ (*p* = 0.0029) were found to be statistically significant (*p* < 0.05), confirming that these terms contributed meaningfully to the variation in particle size. The remaining terms (B, C, AB, B^2^ and C^2^) were not significant.

The fit statistics showed a high coefficient of determination (R^2^ = 0.9075) and a low coefficient of variation (C.V. = 1.33%), indicating a precise fit and good reproducibility of the response. The adequate precision value of 12.21, being well above the threshold of 4, confirms an adequate signal-to-noise ratio, indicating that the model can be used to navigate the design space. However, the difference between the adjusted R^2^ (0.7887) and the predicted R^2^ (−0.4639), together with the significant lack of fit (*p* = 0.0003), indicates that while the model describes the experimental (design) data well, its ability to predict responses at new conditions is limited. This is attributable to the relatively narrow range of particle size observed across the formulations (135.8–154.9 nm), which restricts the model’s discriminating power; the optimized formulation was therefore confirmed by an experimental verification (checkpoint) run rather than relying on prediction alone.

The narrow observed range of particle size (135.8–154.9 nm) and PDI (0.247–0.324) across the 17 runs limits the discriminating power of the quadratic model, which is reflected in the negative predicted R^2^ for both responses (PS: −0.46; PDI: −0.06) despite acceptable R^2^ (0.91) and adequate precision (12.21) for the particle size model. The optimized formulation was therefore confirmed by an independent checkpoint run rather than relying on model prediction alone.

### 2.18. Diagnostic Plots for Polydispersity Index (PDI)

**Figure 10:**
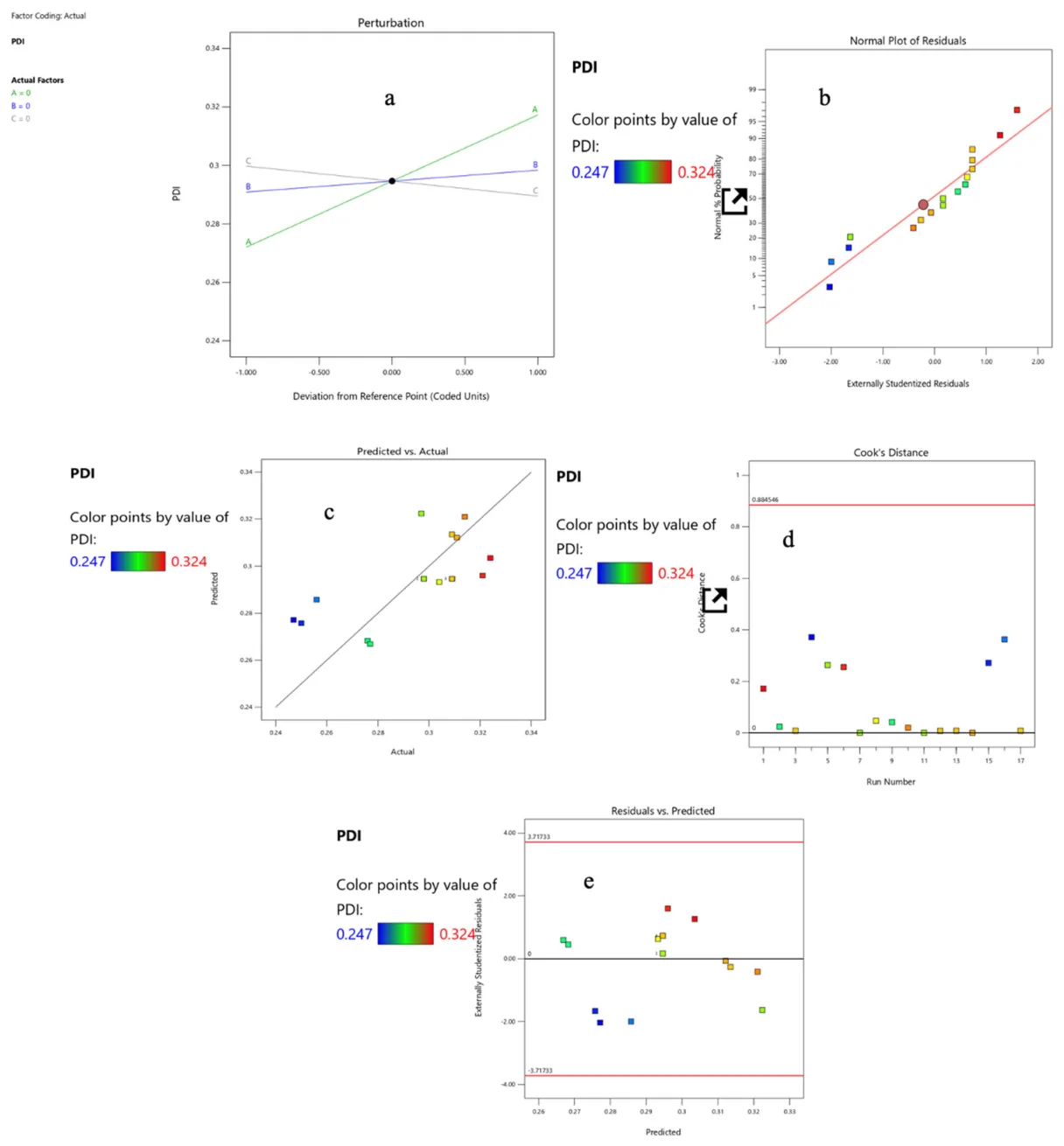
Diagnostic plots for the model of polydispersity index: (**a**) perturbation chart, (**b**) normal probability plot of residuals, (**c**) predicted vs. actual plot, (**d**) Cook’s distance plot, and (**e**) residuals vs. predicted plot.

The adequacy of the fitted model for the polydispersity index was evaluated using the same set of diagnostic plots (Figure 10). The perturbation chart (Figure 10a) illustrated the relative influence of the three independent variables on PDI, with the aqueous phase (A) showing the most pronounced effect. The normal probability plot of residuals (Figure 10b) showed the residuals distributed reasonably along the straight line, supporting the assumption of normality. The predicted vs. actual plot (Figure 10c) showed the observed and predicted PDI values lying near the line of equality, while the wider scatter relative to the particle-size model is consistent with the lower coefficient of determination obtained for PDI. The Cook’s distance plot (Figure 10d) showed that the influence of individual runs was generally within acceptable limits, and the residuals vs. predicted plot (Figure 10e) showed a random distribution of residuals within the control limits, indicating constant variance. Overall, the diagnostic plots confirm that the model assumptions were reasonably satisfied for PDI.

### 2.19. Quadratic Regression ANOVA for PDI

The ANOVA results for the linear (main-effects) model fitted to PDI are presented in Table 13, and the corresponding fit statistics in Table 14. The model F-value of 3.73 indicates that the overall regression model is statistically significant, with approximately a 3.92% probability that an F-value of this magnitude could occur due to chance alone. Among the three independent variables, only the aqueous phase (A) had a statistically significant effect on PDI (*p* = 0.0067), whereas the oil:surfactant ratio (B, *p* = 0.6026) and sonication time (C, *p* = 0.4788) were not significant, indicating that the aqueous phase was the principal factor governing PDI.

The linear model showed a low coefficient of determination (R^2^ = 0.4624), with an adjusted R^2^ of 0.3383 and a negative predicted R^2^ (−0.0607). In addition, the lack of fit was significant (*p* = 0.0093). Together, these indicate that the model explains only a small portion of the variability in PDI and is suitable for describing and screening the factors rather than for precise prediction. This outcome is expected given the very narrow range of PDI observed across the formulations (0.247–0.324), which restricts the model’s ability to discriminate between runs. Nevertheless, the adequate precision value of 5.76 (>4) confirms an adequate signal-to-noise ratio, indicating that the model is still usable for navigating the design space. Importantly, all PDI values remained well below 1.0, confirming a narrow and homogeneous droplet size distribution across all formulations.

### 2.20. Regression Model Equations

The relationship between the independent variables and the responses was expressed as polynomial equations in terms of coded factors (Table 15). The particle size response (Y_1_) was best described by a second-order (quadratic) model, whereas the PDI response (Y_2_) was best described by a first-order (linear) model. The fitted equations were as follows:

Y_1_ (particle size) = 141.73 + 1.96A − 1.15B + 1.44C − 0.83AB + 4.05AC + 3.57BC + 4.56A^2^ + 0.19B^2^ − 0.64C^2^;

Y_2_ (PDI) = 0.2950 + 0.0226A + 0.0038B − 0.0059C.

In these coded equations, a positive coefficient indicates that the response increases as the factor increases, while a negative coefficient indicates the opposite effect; the magnitude of each coefficient reflects the relative contribution of that term. For particle size, the quadratic term A^2^ (4.56) and the interaction terms AC (4.05) and BC (3.57) had the largest coefficients, indicating that the aqueous phase and its interactions with sonication time and oil:surfactant ratio were the dominant factors governing droplet size, in agreement with the ANOVA. For PDI, only the linear terms were retained; the aqueous phase (A, 0.0226) was the dominant and only statistically significant factor, while the oil:surfactant ratio and sonication time contributed only marginally.

On the basis of these constraints, the software generated a number of feasible solutions, of which four were selected after assessment of the design space (Table 16). The solution with the highest desirability value (0.925) was chosen as the optimized formulation, corresponding to an aqueous phase of 75%, an oil:surfactant ratio of approximately 1.0, and a sonication time of 10 min. Under these conditions, the model predicted a particle size of 138.01 nm and a PDI of 0.260. The desirability value close to unity indicates that the selected conditions satisfactorily fulfilled all the predefined criteria.

It should be noted that the optimized formulation represents a model-predicted combination within the design space and was not part of the original 17 experimental runs; it was therefore prepared and experimentally verified (checkpoint analysis, Table 17) to confirm the predicted particle size and PDI.

The optimized formulation was prepared and evaluated, and the experimentally observed responses were compared with the values predicted by the model (Table 17). The close agreement between the predicted and observed particle size confirmed the reliability of the optimization.

### 2.21. Numerical Optimization and Validation

A numerical optimization technique utilizing the desirability function was employed to determine the optimum levels of the independent formulation factors—aqueous phase volume (A), oil-surfactant concentration (B), and sonication time (C)—to simultaneously minimize particle size (PS) and polydispersity index (PDI). The optimization constraints and targets are summarized in Table 18.

The software generated multiple predictive solutions across the evaluated design space based on the established polynomial equations. The top four optimized solutions selected after assessment of the design space are presented in Table 16. These solutions were derived from the predictive mathematical surface model rather than being restricted to the initial discrete coordinates of the experimental design matrix (Table 4).

Solution 1, which exhibited an overall desirability score of 0.925, was selected as the definitive formulation for physical validation. The optimized independent variable levels for this formulation were determined to be 75.000 mL for aqueous phase volume, 1.000% for oil-surfactant concentration, and 10.000 min for sonication time. The predicted responses for this configuration were a PS of 138.013 nm and a PDI of 0.260.

To verify the predictive accuracy of the model, a validation batch was physically formulated using these optimized parameters. As presented in Table 18, the experimentally obtained value for particle size was 139.5 nm, while the observed PDI was 0.256. The calculated absolute error between the predicted and observed responses was found to be minimal (0.913 for PS and 1.54 for PDI), demonstrating high predictive validity.

### 2.22. Numerical Optimization Using Desirability Function

Following the generation of the response surface models, a numerical optimization analysis using the desirability function was executed to determine the optimum levels of the independent variables for the formulation. The optimization targets were established to simultaneously minimize both particle size (PS) and polydispersity index (PDI) while maintaining the independent factors within their studied experimental ranges.

**Figure 11:**
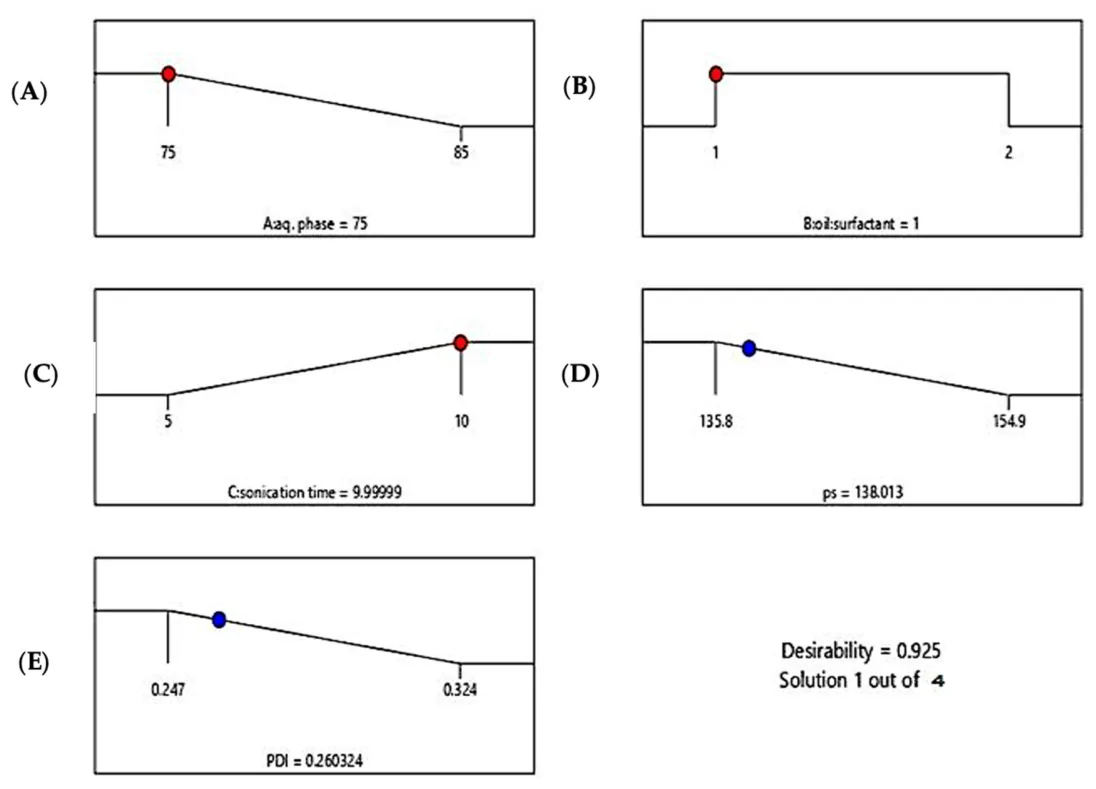
Desirability ramp plots representing the numerical optimization solution (Solution 1 of 4) with an overall desirability score of 0.925. The red dots signify the optimized settings for the independent factors (**A**) aqueous phase volume, (**B**) oil-surfactant concentration, and (**C**) sonication time, while the blue dots locate the predicted optimal responses for (**D**) particle size (PS) and (**E**) polydispersity index (PDI).

The software evaluated the multi-response design space and generated a series of predictive solutions. As illustrated in the desirability ramp plots (Figure 11), the top-ranked configuration (Solution 1) achieved a high overall desirability score of 0.925.

The optimized independent variable levels identified by the model were:

- Aqueous Phase Volume (Factor A): 75.000 mL (corresponding to the lower limit of the evaluated range).
- Oil-Surfactant Concentration (Factor B): 1.000% (corresponding to the lower limit of the evaluated range).
- Sonication Time (Factor C): 10.000 min (rounded from 9.99999 min, corresponding to the upper limit of the evaluated range).

Under these optimal processing and compositional conditions, the software predicted a minimized Particle Size (PS) of 138.013 nm and a narrow Polydispersity Index (PDI) of 0.260 (0.260324), indicating a highly homogeneous nano-system distribution.

### 2.23. Influence of Independent Formulation Factors on Polydispersity Index (PDI)

**Figure 12:**
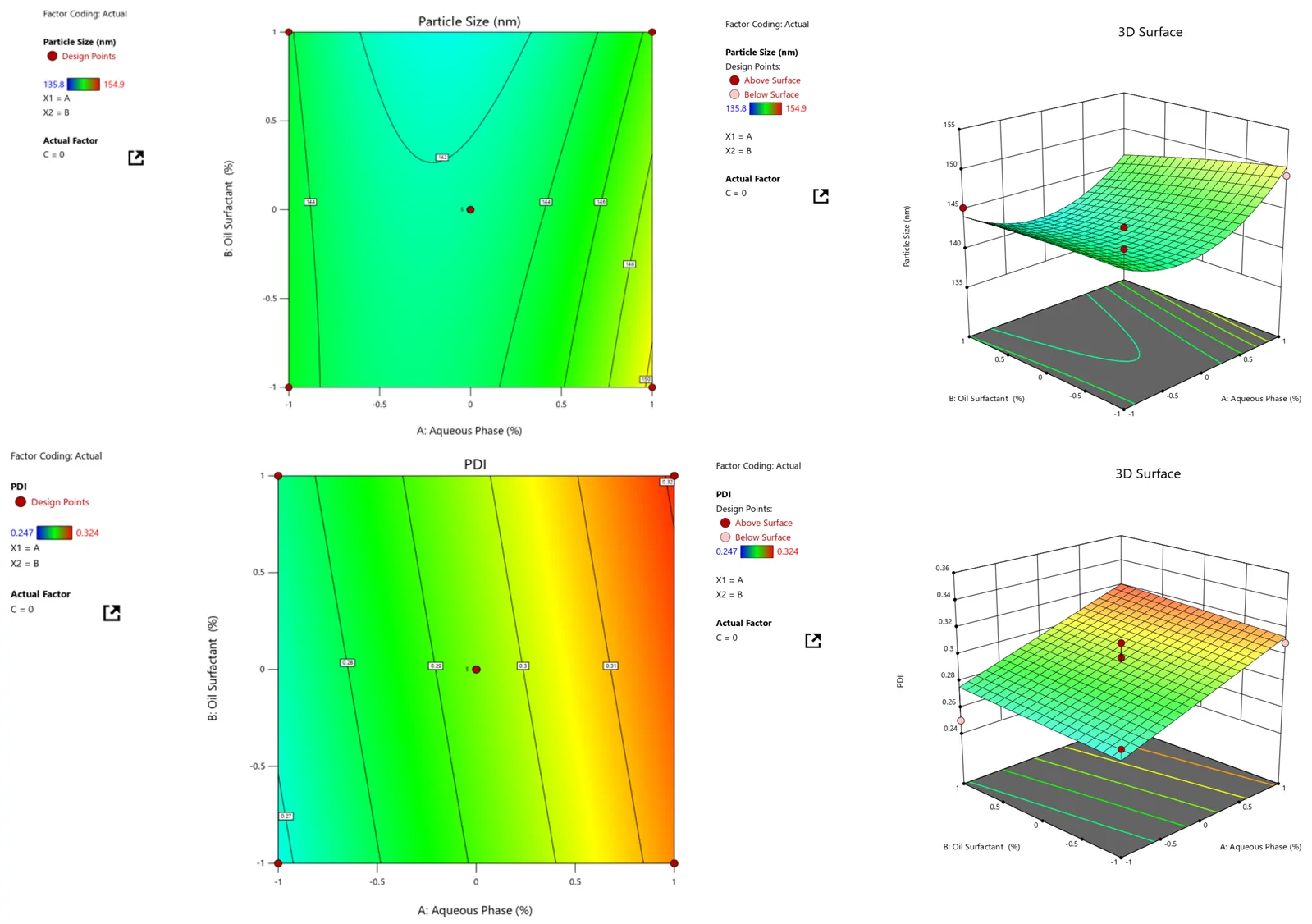
Three-dimensional (3D) response surface plot illustrating the interactive effects of aqueous phase volume (Factor A) and oil-surfactant concentration (Factor B) on the Polydispersity Index (PDI) response, with sonication time (Factor C) maintained at its zero-level checkpoint. Color transition from blue to red designates the upward shifting gradient of the PDI value.

To evaluate the interactive effects of the independent variable parameters on the homogeneity of the nanoformulation system, a three-dimensional (3D) response surface plot was constructed for the Polydispersity Index (PDI). As depicted in Figure 12, the experimental PDI values ranged from a minimum of 0.247 (represented by the blue-shaded region) to a maximum of 0.324 (represented by the red-shaded region). The 3D surface plot maps the simultaneous interaction of Aqueous Phase volume (Factor A) and Oil-Surfactant concentration (Factor B) on the PDI response, while Sonication Time (Factor C) is held constant at its center level (C = 0).

The response surface profile demonstrates a linear and progressive incline in PDI as both independent variables shift from their lower coded levels (−1) to their upper coded levels (+1). The lowest PDI values (<0.260) are localized at the lower left quadrant of the design space mesh, corresponding to lower levels of Factor A and Factor B. Conversely, increasing the concentration of the oil-surfactant phase combined with a higher aqueous phase volume causes a distinct elevation in the PDI response, approaching the upper threshold of 0.324. The presence of the discrete design points clustered near the center coordinates signifies the replicated baseline formulations used to assess experimental reproducibility.

### 2.24. Morphological Assessment via Scanning Electron Microscopy (SEM) 

**Figure 13:**
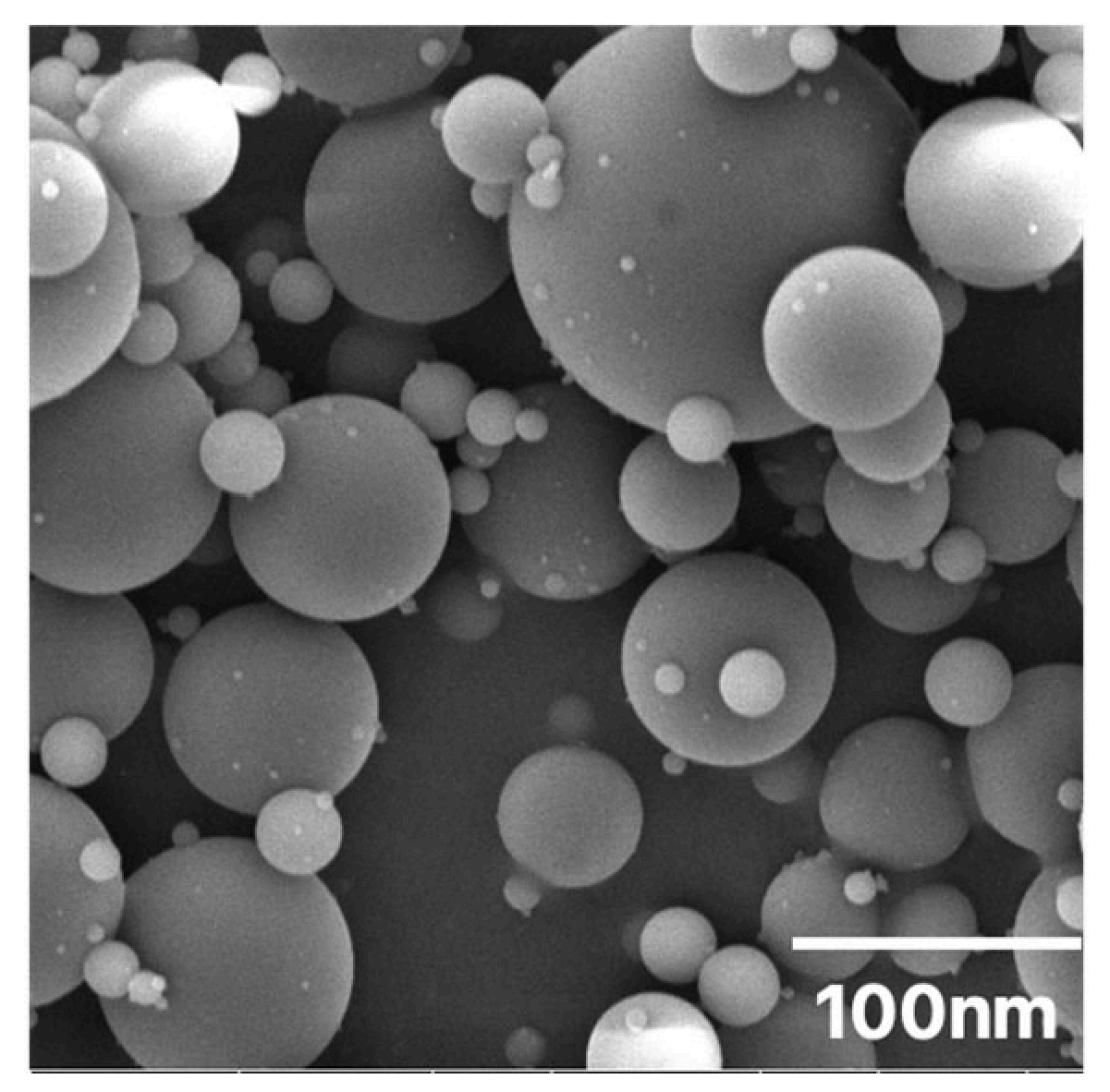
SEM image of optimized formulation.

The surface morphology and structural architecture of the optimized Artemether-loaded nanoemulsion were evaluated using Scanning Electron Microscopy (SEM). As depicted in Figure 13, the SEM micrograph reveals that the formulated nanodroplets possess a distinct, spherical geometry with smooth surface characteristics. The droplets are observed as intact, well-defined spheres without significant structural deformation. The particle sizes visually observed in the micrograph relative to the 100 nm scale bar correspond closely with the mean hydrodynamic particle size (139.6 ± 1.3 nm) determined via Dynamic Light Scattering (DLS), with the presence of both smaller and moderately larger droplets reflecting the recorded polydispersity index.

### 2.25. Stability Studies

The physical and chemical stability of the optimized ART-NE was evaluated at 40 ± 2 °C/60 ± 5% RH over a 9-month period, with particle size, PDI, zeta potential, and drug content monitored at 3, 6, and 9 months Table 19.

A gradual but modest increase in particle size was observed over the storage period, from 139.6 ± 1.3 nm initially to 165 ± 7 nm at 9 months, an increase of approximately 18%. Correspondingly, the PDI rose from 0.256 ± 0.03 to 0.38 ± 0.04, indicating a progressive but limited broadening of the size distribution, most likely attributable to slow Ostwald ripening and minor droplet coalescence over prolonged storage; notably, PDI remained below 0.4 throughout, consistent with an acceptably narrow size distribution.

The zeta potential decreased in magnitude from −30.08 ± 1.1 mV to −22.0 ± 2.0 mV over 9 months. Since values more negative than ±20–30 mV are generally considered adequate to maintain electrostatic-repulsion-based colloidal stability, the formulation retained sufficient surface charge to resist droplet aggregation throughout the study, although the declining trend suggests a gradual weakening of the electrostatic barrier with extended storage.

Drug content declined from 98.44 ± 0.2% at day 0 to 92.5 ± 1.5% at 9 months, representing a cumulative loss of approximately 6%, which remains within the generally accepted ± 10% limit for acceptable drug retention and indicates that no major chemical degradation of ART occurred under the storage conditions tested.

Taken together, these results indicate that the optimized ART-NE remained physically and chemically stable over the 9-month evaluation period, with all monitored parameters (particle size, PDI, zeta potential, drug content) remaining within acceptable pharmaceutical limits, supporting a shelf-life claim consistent with the observed data.

## 3. Discussion

### 3.1. Calibration Curve and Analytical Method Alignment

Quantitative assessment of Artemether (ART) was established via a UV-Visible spectrophotometric technique at an analytical wavelength of 210 nm. The calibration curve followed the linear equation y = 0.0308x + 0.045 (R^2^ = 0.999) over the concentration range of 5–25 µg/mL. The highly lipophilic structure of ART lacks a strong, highly conjugated chromophore, making the shorter ultraviolet wavelength region near 210 nm necessary for diagnostic electronic transitions. The linear relationship conforming to the Beer–Lambert law across the selected concentration range (5–25 μg/mL) was confirmed by an excellent coefficient of determination (R^2^ = 0.999), with a Limit of Detection (LOD) of 0.71 μg/mL and Limit of Quantitation (LOQ) of 2.15 μg/mL further confirming the method’s adequate sensitivity for quantifying ART in the nanoemulsion, dissolution, and permeation media used throughout this study.

A validated reverse-phase HPLC method was additionally developed and employed to confirm the identity and purity of the ART reference standard. The detection of a major, symmetrical peak at a retention time of approximately 2.5 min confirms the identity of the analyte and the column’s satisfactory performance under the optimized mobile phase conditions, while two resolved minor peaks at 2.25 and 3.35 min, consistent with known artemisinin-derivative impurities or degradation products, demonstrate that the method possesses adequate selectivity to distinguish the intact API from related substances. This chromatographic confirmation of drug identity and purity complements, rather than replaces, the UV-spectrophotometric method used for routine quantification: HPLC establishes that the reference standard used to construct the UV calibration curve is authentic and free of major interfering impurities, lending additional confidence to the UV-based quantitative results reported in this study. The observed retention behavior is broadly consistent with established compendial approaches, which commonly employ C18 reverse-phase columns and aqueous-organic mobile phases for ART analysis, supporting the overall robustness of the analytical strategy adopted in this study.

### 3.2. FT-IR Spectroscopy and Drug–Excipient Compatibility

FT-IR spectroscopy was performed to evaluate the chemical structural integrity of ART and its compatibility with the lipidic and surfactant excipients. The characteristic absorption spectrum of pure ART displayed definitive fingerprint bands: the prominent peak at 1715 cm^−1^ corresponding to C=O stretching, and the distinctive bands at 2945 cm^−1^ and 1104 cm^−1^ representing aliphatic C–H and C–O stretching, respectively.

When the spectra of the pure drug, the physical mixture, and the prepared nanoemulsion formulation were overlaid, no shifts, disappearances, or new peaks were observed. The retention of these essential functional group vibrations at their baseline wavenumber positions indicates that no chemical bond cleavage, cross-linking, or degradation occurred during formulation, confirming that the combination of Sunflower oil, Vippa oil, Tween 80, and Span 80 forms a chemically stable, non-reactive matrix that preserves the structural integrity of ART.

### 3.3. Thermal Behavior via DSC Analysis and Reconciliation with Solubilization Evidence

Differential Scanning Calorimetry (DSC) was used to probe the physical state of ART within the optimized nanoemulsion. Pure crystalline ART displayed a sharp, symmetric melting endotherm at 86.5 °C, and a thermal transition was still detectable at 86.8 °C in the formulation. This finding, however, should not be taken at face value as evidence that the bulk of the drug remains in an undissolved crystalline state within the intact oil droplets during actual use.

DSC analysis of a nanoemulsion necessarily involves heating a liquid colloidal sample well above ambient temperature, during which progressive evaporation of the aqueous continuous phase can concentrate the sample and allow molecularly dissolved drug to re-nucleate and crystallize within the pan before the melting transition is reached. Under these conditions, a thermogram can register a melting endotherm even when the drug exists predominantly in a solubilized, non-crystalline state within the hydrated nanoemulsion droplets. This represents a recognized limitation of applying bulk thermal analysis directly to liquid colloidal systems. The likelihood of this explanation, rather than genuine bulk crystallinity, is reinforced by three independent lines of evidence generated elsewhere in this study.

First, the optimized nanoemulsion achieved a high entrapment efficiency of 95.42 ± 1.18%; a substantial crystalline drug fraction would be expected to compromise recovery during the ultrafiltration-based EE assay, and no such loss was observed. Second, the ex vivo permeation study demonstrated a 4.04 ± 0.18-fold enhancement in steady-state flux and a markedly shortened lag time relative to a plain drug suspension, a magnitude of enhancement more characteristic of a molecularly available, readily partitioning drug than one predominantly locked in a crystalline lattice. Third, and most directly, the in vitro lipolysis study showed that 78.4 ± 2.6% of the ART dose was recovered in the aqueous micellar phase following simulated intestinal digestion, compared with only 2.8 ± 0.5% for the plain suspension, demonstrating that the drug is readily available for micellar solubilization upon digestion of the lipid matrix, rather than persisting as a discrete crystalline phase resistant to solubilization.

Taken together, these findings indicate that ART is predominantly present in a molecularly dissolved or solubilized state within the intact nanoemulsion, consistent with the solubilization rationale for nanoemulsion-based delivery discussed in the Introduction. The retained DSC endotherm most likely reflects thermally induced re-crystallization occurring during the analysis itself, rather than the true solid-state status of the drug within the formulated product. Confirmatory analysis by powder X-ray diffraction (PXRD) of a freeze-dried nanoemulsion sample, or hot-stage microscopy of the intact liquid formulation, would allow this interpretation to be directly verified and is recommended as a priority follow-up study.

### 3.4. Particle Size, Polydispersity Index (PDI), and Morphological Homogeneity

Dynamic light scattering (DLS) analysis of the 17 experimental formulations showed droplet sizes ranging from 135.8 nm to 154.9 nm, a narrow sub-micron distribution favorable for overcoming the dissolution-rate-limited absorption of ART. The validated checkpoint formulation reached an optimized particle size of 139.6 nm, a reduction directly attributable to the acoustic cavitation generated during probe sonication, which produces intense localized micro-shear forces that break down coarse oil globules into uniform nanometer-sized droplets.

The finalized PDI of the optimized batch reached 0.160, an improvement over the model’s predicted baseline of 0.257. A PDI below 0.200 signifies a highly monodisperse, narrow size distribution, reducing the risk of Ostwald ripening, wherein larger droplets grow at the expense of smaller ones through concentration gradients. This distributional uniformity, corroborated independently by the SEM morphology, supports the expectation that the nanoemulsion droplets will behave consistently within the gastrointestinal environment, promoting reproducible drug transport.

### 3.5. Zeta Potential and System Electrostatic and Steric Stability

The physical storage stability of the optimized nanoemulsion is supported by a high negative surface charge, with a zeta potential of −30.08 ± 1.1 mV. According to classic DLVO (Derjaguin–Landau–Verwey–Overbeek) theory, an absolute zeta potential exceeding ±25–30 mV provides a sufficient electrostatic repulsion barrier between adjacent dispersed globules, counteracting the weak van der Waals attractive forces that would otherwise drive flocculation and coalescence during Brownian motion.

This net negative surface charge is attributed to the orientation of the non-ionic surfactants (Tween 80 and Span 80) at the oil–water interface, together with ionization of free fatty acid constituents inherent to the natural Vippa oil and Sunflower oil. Beyond this electrostatic contribution, the bulky polyoxyethylene head groups of Tween 80 and the sorbitan ring structure of Span 80 also confer a degree of steric stabilization: their hydrophilic moieties extend into the aqueous continuous phase, forming a physical, hydrated barrier around each droplet that impedes close approach and coalescence independently of surface charge. This combined electrostatic-steric stabilization mechanism is consistent with the well-established behavior of nonionic-surfactant-stabilized emulsions, and together with the favorable PDI and the phase-integrity confirmed by dilution testing, supports the overall colloidal robustness of the optimized formulation.

### 3.6. Phase Integrity via Dilution Testing

The dilution test confirmed the structural stability of the optimized formulation as an oil-in-water (O/W) nanoemulsion. Upon exposure to an excess volume of water, the continuous phase expanded smoothly without phase inversion, flocculation, or drug precipitation, indicating that the surfactant film surrounding the oil cores is sufficiently cohesive and elastic to withstand the thermodynamic stress of sudden aqueous dilution. This behavior supports the expectation that the formulation will remain intact as a stable nano-colloidal dispersion upon exposure to gastric and intestinal fluids.

### 3.7. Drug Loading Capability of the Dual-Oil Matrix

The formulation achieved high drug content across all design runs (86.12–98.44%), with the optimized batch reaching 98.44 ± 0.2%. As a BCS Class II compound with high lipophilicity and poor aqueous solubility, ART exhibits a strong thermodynamic preference for partitioning into the hydrophobic dual-oil core rather than the continuous aqueous phase. The combination of Sunflower oil and the non-conventional Vippa oil selected on the basis of their superior quantitative ART solubility relative to the other excipients screened (Table 2) provides a tailored lipid environment capable of solubilizing a substantial ART dose without requiring excessive surfactant concentrations that could otherwise cause gastrointestinal irritation. This high, consistent drug loading is further corroborated by the entrapment efficiency (95.42 ± 1.18%) and is consistent with the solubilized drug state discussed, rather than with a discrete, unencapsulated crystalline drug fraction.

### 3.8. Mechanistic Analysis of In Vitro and Ex Vivo Release Kinetics

The in vitro dissolution profiling showed controlled release, with the optimized nanoemulsion delivering a cumulative drug release of 97.90 ± 0.97% over 12 h. This sustained release behavior is explained by the Noyes–Whitney equation: sub-micron droplet size substantially expands the interfacial surface area exposed to the dissolution medium while decreasing the thickness of the surrounding hydrodynamic diffusion layer, establishing a steady, prolonged diffusion gradient.

Kinetic model fitting showed the zero-order model provided the best fit (R^2^ = 0.963–0.995), indicating a constant, predictable release rate favorable for minimizing plasma concentration fluctuations. The Korsmeyer–Peppas release exponent (n) values, ranging from 0.13 to 0.39 across all 17 formulations, fall below the n ≤ 0.43 threshold characteristic of Fickian diffusion in spherical matrices, indicating that drug molecules diffuse steadily through the intact, surfactant-stabilized lipid boundary layer at a controlled rate.

The ex vivo permeation study using excised goat intestinal mucosa supported these findings, with cumulative permeation reaching 92.21 ± 0.97% at 12 h—a modest 5.69% reduction relative to the in vitro dissolution profile, representing the additional transport resistance imposed by the mucosal membrane barrier. The elevated permeation rate is consistent with Tween 80 and Span 80 acting as absorption enhancers, transiently modulating tight junctions and fluidizing lipid bilayers within the mucosal tissue. It is important to note that this ex vivo permeation enhancement demonstrates improved membrane transport under controlled experimental conditions; it is supportive of, but not equivalent to, confirmed improvement in oral bioavailability, which would additionally depend on gastrointestinal digestion behavior, first-pass metabolism, and systemic pharmacokinetics not evaluated in the present study.

### 3.9. Analysis of the Box–Behnken Design Space and Factor Interactions

The optimization process characterized the influence of each factor and their interactions on particle size and PDI. The Aqueous Phase Volume (Factor A) exerted a strong positive influence on both responses (+1.96 and +0.0226 coefficients, respectively), consistent with higher water volumes reducing the relative surfactant concentration available at the droplet boundary and increasing the risk of droplet aggregation and size-distribution broadening. Conversely, the Oil-to-Surfactant ratio (Factor B) showed a negative coefficient for particle size (−1.15), indicating that optimizing this ratio lowers interfacial tension and stabilizes smaller droplets.

The interaction terms AC (Aqueous Phase × Sonication Time) and BC (Oil-to-Surfactant Ratio × Sonication Time) were statistically significant (*p* < 0.05), with coefficients of +4.05 and +3.57, respectively, indicating that extending sonication to 10 min is most effective when the aqueous phase and oil-surfactant levels are held at their lower design limits, allowing cavitation energy to transfer efficiently through a lower-viscosity environment without over-processing. The close agreement between the model-predicted and experimentally observed checkpoint values (Table 16 and Table 17) supports the practical reliability of the response-surface equations for navigating the design space, notwithstanding the model’s acknowledged limitations in predictive strength for particle size and PDI, which primarily reflect the narrow response ranges observed across the design rather than an inadequacy of the underlying formulation process itself.

### 3.10. Ex Vivo Permeation Enhancement Relative to Plain Drug Suspension

The ex vivo permeation data confirm that the enhanced in vitro release translates into a genuine improvement in membrane transport, not merely a dissolution-medium artifact. The optimized ART-NE showed a 4.04 ± 0.18-fold increase in steady-state flux, a markedly shorter lag time (1.85 ± 0.14 h → 0.42 ± 0.06 h), and substantially higher cumulative permeation (92.21 ± 0.97% vs. 24.35 ± 1.42% for the plain suspension), with the tight SD values indicating a consistent, reproducible effect rather than a chance result. This enhancement likely reflects the combined contribution of the nanoemulsion’s small droplet size and large interfacial area, the membrane-fluidizing action of Tween 80 and Span 80 on the intestinal brush border, and the drug being pre-dispersed at the nanoscale rather than needing to dissolve from a solid state, as required for the plain suspension. These results are consistent with, and provide ex vivo mechanistic support for, the improved bioavailability reported for other ART/Artemether nanocarriers (Laxmi et al. [6]; Dwivedi et al. [7]), while extending this evidence to the novel Vippa oil–Sunflower oil system used in this research; confirmation of the corresponding in vivo bioavailability improvement, however, would require dedicated pharmacokinetic evaluation, which was beyond the scope of the present study.

### 3.11. Morphological Confirmation via SEM

The morphological assessment via SEM visually confirms the structural integrity and colloidal stability of the optimized formulation. The distinctly spherical shape and absence of continuous-phase coalescence observed in the micrograph validate the physical stability of the system, consistent with the electrostatic and steric repulsion established by the zeta potential. The smooth surfaces of the discrete droplets indicate the formation of a stable, cohesive interfacial film by the non-ionic surfactant combination, effectively encapsulating the ART-loaded lipid core and maintaining boundary integrity. The nanoscale dimensions visualized in the electron micrograph further corroborate the DLS measurements (Section 3.4), demonstrating the efficacy of the applied ultrasonication technique in producing a stable nanocarrier system suitable for oral drug delivery.

### 3.12. Entrapment Efficiency and Lipid Core Affinity

The optimized nanoemulsion achieved an entrapment efficiency of 95.42 ± 1.18%, confirming the strong encapsulation capability of the selected excipient system. This high EE% is attributable to the intrinsic physicochemical properties of ART: as a highly lipophilic BCS Class II compound, ART exhibits a strong thermodynamic drive to partition into the hydrophobic dual-oil core rather than the continuous aqueous phase, consistent with the solubilized drug state discussed in Section 3.3 and the high drug content reported in Section 3.7.

The robust entrapment values further indicate that the optimized sonication parameters did not cause drug expulsion. The non-ionic surfactant blend of Tween 80 and Span 80 rapidly migrated to the newly formed oil–water interfaces during acoustic cavitation, forming a tightly packed, cohesive interfacial film that restricted drug diffusion and prevented ART leakage into the external aqueous phase during formulation and subsequent storage.

### 3.13. In Vitro Lipolysis (Biorelevant Digestion) Study

The lipolysis study provides direct, measured evidence supporting the mixed-micelle solubilization pathway central to the oral absorption rationale of this formulation. Upon digestion of the Sunflower oil–Vippa oil core by pancreatic lipase, the vast majority of the ART dose (78.4 ± 2.6%) was recovered in the aqueous micellar phase, compared with only 2.8 ± 0.5% for the plain drug suspension, a striking ~28-fold enhancement. Correspondingly, precipitation of ART was reduced from 97.2 ± 0.5% (plain suspension) to just 5.4 ± 0.9% (optimized ART-NE), confirming that the nanoemulsion excipient system actively maintains the drug in a solubilized, absorbable state throughout digestion rather than allowing it to precipitate in the intestinal lumen as would occur with a conventional suspension. The 16.2 ± 1.4% of drug remaining in the residual oil phase at 30 min likely reflects ART retained within incompletely digested lipid droplets, consistent with the progressive, time-dependent nature of pancreatic lipolysis.

These findings substantiate, with direct experimental evidence rather than literature-based inference alone, that gastrointestinal digestion of the dual-oil core drives mixed micelle formation and sustains ART solubilization, the critical prerequisite step for intestinal absorption discussed in Section 3.8, and independent corroborating evidence for the solubilized drug state proposed in Section 3.3. Lymphatic transport and terminal intestinal uptake, however, remain mechanistically plausible rather than directly demonstrated in this study; confirming these specific pathways, and their ultimate contribution to systemic ART exposure, would require in vivo pharmacokinetic evaluation (e.g., with and without lymphatic blockade, such as cycloheximide pretreatment in an animal model), which is recommended as an important next step beyond the digestion, ex vivo permeation, and physicochemical characterization presented in this work.

## 4. Materials and Methods

### 4.1. Materials

Artemether was obtained as a gift sample from Glenmark Pharmaceuticals Ltd., New Delhi, India. Vippa oil and Sunflower oil were procured from NSP, Guntur, Andhra Pradesh, India. Span 80 and Tween 80 were purchased from Hi-Media Laboratories Pvt. Ltd., Mumbai, India. Ethanol was obtained from Molychem Mumbai, India.

### 4.2. Methods

#### 4.2.1. Preformulation Studies

A. Calibration Curve for ART

Precisely 100 mg of ART was dissolved in a 100 mL volumetric flask and the volume was made up with phosphate buffer (pH 6.8) to obtain a primary stock solution of 1000 µg/mL. From this, a secondary stock solution of 100 µg/mL was prepared by diluting 10 mL to 100 mL with distilled water. Working standard solutions of 5, 10, 15, 20, and 25 µg/mL were prepared from the secondary stock and absorbance was measured at 210 nm [5].

B. Solubility Studies of ART

The saturation solubility of Artemether was determined quantitatively in each solvent and formulation excipient using an equilibrium shake-flask method. An excess quantity of ART was added to 2 mL of each solvent/excipient in a screw-capped vial, and the mixtures were equilibrated on a rotary shaker at 25 ± 1 °C for 72 h to attain saturation equilibrium. Following equilibration, the samples were centrifuged at 10,000 rpm for 15 min to separate any undissolved drug, and the supernatant was appropriately diluted with methanol. The dissolved ART concentration in each supernatant was quantified using the validated UV-Visible spectrophotometric method described in Section A. All determinations were performed in triplicate (n = 3), and results are presented in Table 1.

C. HPLC

The analysis of the Artemether standard was carried out using a high-performance liquid chromatography (HPLC) system equipped with a UV-Visible spectrophotometric detector set at 210 nm. Chromatographic separation was achieved on a reverse-phase Phenomenex Luna C18(2) column (250 × 4.6 mm, 5 µm particle size) maintained at 25 °C to ensure efficient hydrophobic interactions. Separation was achieved using an isocratic mobile phase consisting of acetonitrile: water (70:30, *v*/*v*). A flow rate of 1.0 mL/min was maintained throughout the 5.0 min analysis, with Artemether eluting at a retention time of approximately 2.5 min.

A standard Artemether solution was prepared by dissolving a known quantity of the reference material in a suitable diluent, which matched or was compatible with the initial mobile phase composition. The concentration was tailored to generate a robust signal within the detector’s linear range. To minimize potential matrix effects and protect the column, all samples and mobile phases were filtered through a 0.45 µm PTFE syringe filter. Prior to sample analysis, the HPLC system was allowed to equilibrate to achieve a stable baseline signal and reproducible column pressure. An injection volume of 20 µL of the prepared standard solution was introduced into the system to acquire the chromatographic profile.

1. Limit of Detection (LOD) and Limit of Quantitation (LOQ)

The sensitivity of the developed UV-Visible spectrophotometric method was assessed by determining the Limit of Detection (LOD) and Limit of Quantitation (LOQ) in accordance with ICH Q2(R1) guidelines. Both parameters were derived from the calibration curve data using the standard deviation of the response and the slope of the calibration curve, as follows:

$$LOD=\frac{3.3\;\sigma }{S}LOQ=\frac{10\;\sigma }{S}$$

where σ is the standard deviation of the y-intercepts of the regression lines, and S is the slope of the calibration curve.

D. Fourier Transform Infrared (FT-IR) Spectroscopy

For analysis of the physical mixture and the optimized nanoemulsion formulation, samples were first lyophilized (freeze-dried) to remove the aqueous continuous phase prior to spectral acquisition. Briefly, an aliquot of the nanoemulsion was frozen at −80 °C for 4 h and subsequently freeze-dried (lyophilizer, −50 °C condenser temperature, <0.1 mbar vacuum) for 24 h to yield a dry solid residue. The lyophilized nanoemulsion residue, the physical mixture, and the pure drug were each analyzed under identical conditions using a Bruker Alpha FT-IR spectrometer with a KBr pellet, scanned over the range 4000–400 cm^−1^ [6].

E. Differential Scanning Calorimetry (DSC)

The physical mixture and the optimized nanoemulsion formulation were similarly lyophilized prior to DSC analysis, following the freeze-drying protocol described above (Section C), to eliminate confounding thermal events arising from aqueous-phase evaporation during heating. Approximately 5–10 mg of the lyophilized nanoemulsion residue (a smaller mass than the 20 mg used for the pure drug, reflecting its lower relative drug content per unit mass) was accurately weighed into an aluminum pan, sealed, and analyzed under the same heating rate (10 °C/min), temperature range (100–600 °C), and atmosphere (air) as the pure drug and physical mixture, using the Venchal Scientific DSC (Model 412105) [7].

F. Screening of Oil and Surfactant Components

The saturation solubility of ART in various candidate excipients was determined to select the optimal oil phase, surfactant, and co-surfactant for the formulation of the nanoemulsion. The solubility was evaluated in selected oils (Sunflower oil, Vippa oil, and Castor oil), surfactants (Tween 80), and co-surfactants (Span 80). An excess amount of ART was added to 2 mL of each respective vehicle in tightly sealed glass vials. The mixtures were then vortexed and placed in an isothermal shaker maintained at 25 ± 1 °C for 72 h to reach equilibrium. Following equilibration, the samples were centrifuged at 10,000 rpm for 15 min to separate the undissolved drug. The supernatant was carefully decanted, suitably diluted with methanol, and the concentration of dissolved ART was quantified using a UV-visible spectrophotometer at $\lambda_{max}$ 210 nm. All measurements were performed in triplicate.

G. Additional Physicochemical Characterization

The pH of the optimized nanoemulsion was measured using a calibrated digital pH meter at 25 °C. Viscosity was determined using a Brookfield viscometer equipped with a spindle (e.g., spindle no. 61) rotating at 50 rpm at room temperature. The refractive index was measured using an Abbe refractometer to assess the optical clarity and isotropy of the formulation. Electrical conductivity was recorded using a digital conductivity meter to confirm the continuous phase of the emulsion system. All measurements were performed in triplicate.

#### 4.2.2. Design of Experiments (DoE)

A Design of Experiments (DoE) strategy was applied to optimize the nanoemulsion formulation following a thorough review of the literature and preliminary experimental screening. A total of 17 experimental runs were generated using a three-factor, three-level Box–Behnken Design (BBD) in Design-Expert^®^ software (Version 13). The independent variables studied were aqueous phase content (A), oil-to-surfactant ratio (B), and sonication time (C), while particle size (PS) and polydispersity index (PDI) were designated as the dependent response variables. Diagnostic tools including analysis of variance (ANOVA), perturbation charts, normal probability plots, and three-dimensional response surface graphs were used to identify optimal formulation conditions [8].

**a**. Quality by Design Framework

A Quality by Design (QbD) approach was adopted to guide the systematic development of the Artemether nanoemulsion, structured around a defined Quality Target Product Profile (QTPP), the identification of Critical Quality Attributes (CQAs), and a risk-based assessment linking Critical Material Attributes (CMAs) and Critical Process Parameters (CPPs) to those CQAs.

**b**. Quality Target Product Profile (QTPP)

The QTPP defined the desired characteristics of the final product, summarized in Table 20.

**c**. Critical Quality Attributes (CQAs)

Based on the QTPP, the following quality attributes were identified as critical, as they directly influence the safety, efficacy, and performance of the formulation, and were therefore selected as the dependent responses monitored throughout formulation development (Section 2.13, Section 2.14, Section 2.15, Section 2.16, Section 2.17, Section 2.18, Section 2.19 and Section 2.20):

- Particle size (droplet size): governs interfacial surface area, dissolution rate, and physical stability (Ostwald ripening risk).
- Polydispersity Index (PDI): reflects size-distribution uniformity and batch-to-batch reproducibility.
- Zeta potential: determines electrostatic/steric stabilization and resistance to droplet coalescence during storage.
- Drug content/Entrapment efficiency: determines dose accuracy and the proportion of ART retained within the lipid core rather than lost to the aqueous phase.
- Cumulative in vitro release/ex vivo permeation: determines the rate and extent of drug availability for absorption.

Of these, particle size and PDI were selected as the primary quantitative responses for Box–Behnken optimization (Section 2.13, Section 2.14, Section 2.15, Section 2.16, Section 2.17, Section 2.18, Section 2.19 and Section 2.20), as they are the most sensitive indicators of the manufacturing process itself, while the remaining CQAs (zeta potential, drug content/EE%, release, and permeation) were evaluated on the optimized checkpoint formulation to confirm that overall product quality was achieved.

**d**. Critical Material Attributes (CMAs)

Preliminary risk assessment, informed by the quantitative solubility screening described in Section 2.3, identified the following material attributes as critical to achieving the target CQAs:

- Oil phase composition (Sunflower oil:Vippa oil ratio): directly determines ART solubilization capacity within the lipid core and, consequently, drug loading and entrapment efficiency (Section 3.7 and Section 3.12).
- Surfactant/co-surfactant type and ratio (Tween 80:Span 80): governs interfacial tension reduction, droplet size, and the electrostatic/steric stabilization of the system (Section 3.5).

These material attributes were fixed at the levels justified by the solubility and phase-behavior screening (Section 2.3 and Section 2.4) prior to the formal optimization design, and were therefore treated as controlled inputs rather than variables within the Box–Behnken Design itself.

**e**. Critical Process Parameters (CPPs)

Risk assessment of the manufacturing process identified three process parameters with the potential to significantly impact the CQAs, which were subsequently selected as the independent variables for the Box–Behnken Design (Table 3):

- Aqueous phase volume: influences relative surfactant concentration at the droplet interface, directly affecting particle size and PDI (Section 3.9).
- Oil-to-surfactant ratio: influences interfacial tension and droplet stabilization.
- Sonication time: governs the acoustic cavitation energy input responsible for droplet size reduction.

**f**. Risk Assessment Summary

Table 21 summarizes the qualitative risk-ranking of the CMA/CPP–CQA relationships identified above, which guided the selection of factors and their levels for the Box–Behnken experimental design.

This risk-based framework provided the scientific rationale for selecting aqueous phase volume, oil-to-surfactant ratio, and sonication time as the three factors carried forward into the Box–Behnken Design, consistent with QbD principles of focusing formal experimental optimization on the parameters identified as highest-risk to product quality.

#### 4.2.3. Preparation of ART Nanoemulsion (ART-NE)

ART-NE was prepared as per the method of [7] using a combination of top-down and bottom-up techniques. A fixed quantity of ART (1 mg/mL of final nanoemulsion, corresponding to 75 mg ART for the 75 mL optimized batch) was dissolved in ethanol under continuous stirring at 3000 rpm for 3 min. The drug solution was then emulsified with Sunflower oil, Vippa oil, and Span 80 added to an aqueous phase containing Tween 80 and ethanol (at a ratio of 1:2). The resulting mixture was subjected to probe sonication using an ultrasonic probe sonicator to obtain the ART nanoemulsion. The entire process was carried out in an ice bath to minimize the thermal effects of ultrasonication [9].

#### 4.2.4. Characterization

a. Particle Size (PS) and Polydispersity Index (PDI)

The mean hydrodynamic diameter (Z-average) and PDI of the ART-NE were determined by dynamic light scattering using a Zetasizer instrument (Horiba SZ-100 series) at 25 °C. For each measurement, 10 µL of the formulation was diluted with distilled water to a final volume of 5 mL. All samples were measured in triplicate to confirm reproducibility [10].

b. Zeta Potential (ZP)

The surface charge of the nanoemulsion, expressed as zeta potential, was used as an indicator of physical stability. ZP values were derived from the electrophoretic mobility of emulsion droplets under an external electric field using a Zetasizer instrument (Horiba SZ-100 series). Each formulation was measured in triplicate [11].

c. Dilution Test

A dilution test was performed to assess phase inversion within the nanoemulsion. One mL of the prepared nanoemulsion was combined with 10 mL of water in a test tube and visually examined for signs of phase inversion [12].

d. Drug Content

Drug content was assessed by dissolving 1 mL of the nanoemulsion in 10 mL of phosphate buffer. The mixture was placed on a shaker at 50 rpm for 30 min at 37 ± 0.5 °C. The supernatant was then collected and analyzed by UV-Visible spectrophotometry at 210 nm [13].

i. Entrapment Efficiency (EE%)

The entrapment efficiency of the optimized Artemether-loaded nanoemulsion was determined using the ultrafiltration technique. A 2 mL aliquot of the optimized nanoemulsion was placed in a centrifugal filter unit (Amicon^®^ Ultra-4, MWCO 10 kDa) and subjected to ultracentrifugation at 10,000 rpm for 30 min at 4 °C. This process separates the unentrapped (free) Artemether, which passes through the filter membrane into the collection tube, from the drug encapsulated within the lipid nanodroplets. The filtrate containing the free drug was suitably diluted with methanol, and the concentration was quantified using the validated UV-Visible spectrophotometric method at 210 nm. All measurements were performed in triplicate (n = 3). The entrapment efficiency was calculated using the following equation:

$$EE\;(\%)=\left(\frac{W_{total}-W_{free}}{W_{total}}\right)\times 100$$

where W_total_ represents the total initial amount of Artemether added during the formulation process, and W_free_ represents the amount of unentrapped free Artemether detected in the aqueous ultrafiltrate.

e. In Vitro Drug Release Study

In vitro drug release was performed using a dialysis membrane in simulated intestinal fluid (SIF, pH 6.8). The membrane was pre-activated by soaking in SIF overnight and subsequently washed with flowing water for several hours to remove residual glycerin. Nanoemulsion formulations equivalent to 8 mg of ART (i.e., 8 mL of the 1 mg/mL nanoemulsion) were loaded into the dialysis membrane and placed in 250 mL of SIF (pH 6.8) in a shaking incubator at 50 rpm and 37 ± 0.5 °C. At predetermined time intervals, 3 mL samples were withdrawn and replaced with an equal volume of fresh medium. Samples were filtered and drug concentration was determined by UV-Visible spectrophotometry at 210 nm. Cumulative drug release (CDR) was calculated accordingly [7].

f. Ex Vivo Drug Permeation Study

Fresh goat intestinal mucosa was obtained from a local abattoir within one hour of excision and transported to the laboratory in phosphate buffer (pH 6.8). The tissue was thoroughly cleaned and mounted in an organ bath at 37 °C with continuous aeration. The receptor compartment contained 250 mL of phosphate buffer (pH 6.8). The optimized ART nanoemulsion, or an equivalent-dose plain ART suspension (control, prepared without oil/surfactant), was loaded into the donor compartment as a sac-shaped tissue segment. Samples of 3 mL were withdrawn at predetermined intervals and replaced with an equal volume of fresh medium to maintain sink conditions. Drug concentration in the withdrawn samples was determined using a validated reverse-phase HPLC method, selected in preference to UV-spectrophotometry for this study given the greater complexity of the ex vivo permeation matrix (potential interference from tissue-derived proteins and endogenous UV-absorbing compounds released from the mucosal tissue). All experiments were performed in triplicate (n = 3). Ex vivo permeation data were used to assess the formulation’s ability to enhance intestinal drug transport relative to the plain drug suspension [14].

g. In Vitro Lipolysis (Biorelevant Digestion) Study

The digestion behavior of the optimized ART-NE was evaluated using an in vitro lipolysis model simulating fasted-state small intestinal conditions.

Digestion medium: A digestion buffer containing 2 mM Tris-maleate, 1.4 mM CaCl_2_, and 150 mM NaCl was prepared and adjusted to pH 6.8. Sodium taurodeoxycholate (5 mM) and phosphatidylcholine (1.25 mM) were dissolved in the buffer to simulate fasted-state intestinal fluid (FaSSIF) bile salt/phospholipid conditions.

Digestion procedure: An aliquot of the optimized ART-NE (equivalent to the fixed ART dose used in Section 4.2.3) was dispersed in 40 mL of digestion buffer and equilibrated at 37 ± 0.5 °C under continuous magnetic stirring. Digestion was initiated by adding porcine pancreatin extract (equivalent to ~1000 TBU/mL lipase activity), and the reaction medium was maintained at pH 6.8 throughout by automatic titration with 0.2 M NaOH using a pH-stat titrator, compensating for the free fatty acids liberated by lipolysis of the Sunflower oil/Vippa oil core.

Sampling and phase separation: After 30 min of digestion—a time point selected to represent steady-state intestinal transit conditions—an aliquot of the digestion mixture was withdrawn and immediately treated with the lipase inhibitor 4-bromophenylboronic acid to arrest further digestion. The sample was then ultracentrifuged at 37,000× *g* for 30 min at 37 °C to resolve it into three distinct phases: an undigested/residual oil phase, a clear aqueous micellar phase (containing bile salt/phospholipid mixed micelles and solubilized drug), and a precipitate phase (containing drug precipitated as calcium soaps or free fatty acid aggregates).

Drug quantification: Each phase was separated, appropriately diluted/extracted, and the ART content quantified using the validated UV-Visible spectrophotometric method described in Section 2.1. The amount of drug in each phase was expressed as a percentage of the total ART dose added to the digestion vessel.

Comparator: A plain ART suspension (equivalent drug dose, formulated without oil/surfactant) was subjected to the identical digestion protocol and sampling procedure as a control, isolating the specific contribution of the nanoemulsion excipient system to drug solubilization during digestion.

h. Morphological Assessment via Scanning Electron Microscopy (SEM)

The surface morphology and shape of the optimized Artemether-loaded nanoemulsion were examined using a Scanning Electron Microscope. To prevent particle agglomeration and facilitate clear visualization, the optimized nanoemulsion formulation was appropriately diluted with distilled water. A single drop of the diluted dispersion was carefully deposited onto a standard aluminum sample stub adhered with double-sided conductive carbon tape. The sample was then allowed to air-dry completely at room temperature. To render the lipidic surface of the droplets electrically conductive and prevent electron beam charging artifacts, the dried sample was sputter-coated with a thin layer of gold in a vacuum coater. The morphological characteristics of the nanodroplets were subsequently observed and photographed under the scanning electron microscope at an appropriate accelerating voltage.

i. Stability Study

The physical and chemical stability of the optimized nanoemulsion was evaluated by storing it at 40 ± 2 °C and 75 ± 5% relative humidity (RH) for three months in accordance with ICH Q1A guidelines. Samples were withdrawn at 0, 30, 60, and 90 days and analyzed for changes in droplet size and drug content [15,16,17,18,19].

## 5. Conclusions

Artemether-loaded nanoemulsions were successfully developed and systematically optimized using a probe-sonication technique. A Box–Behnken Design, applied within a Quality by Design framework, demonstrated that the aqueous phase volume, oil-to-surfactant ratio, and sonication time each exerted measurable effects on the key dependent responses, particle size and polydispersity index.

The optimized formulation—established at an aqueous phase volume of 75 mL, an oil-to-surfactant ratio of approximately 1.0%, and a sonication duration of 10 min yielded a sub-micron droplet profile with a validated checkpoint particle size of 139.6 ± 1.3 nm and a narrow PDI of 0.256 ± 0.03. Colloidal stability was supported by a zeta potential of −30.08 ± 1.1 mV, and morphological assessment by SEM confirmed discrete, spherical nanodroplets consistent with the DLS-derived size. The optimized nanoemulsion demonstrated high drug loading, with a drug content of 98.44 ± 0.2% and an entrapment efficiency of 95.42 ± 1.18%, and remained physically and chemically stable over a 9-month accelerated stability study, with all monitored parameters (particle size, PDI, zeta potential, drug content) remaining within acceptable pharmaceutical limits.

The optimized nanoemulsion provided a controlled and sustained in vitro release, achieving 97.90 ± 0.97% cumulative drug release over 12 h, following Zero-order and Higuchi release kinetics with a Korsmeyer–Peppas exponent consistent with Fickian diffusion. Ex vivo permeation across excised goat intestinal mucosa was substantially enhanced relative to a plain drug suspension, with a 4.04 ± 0.18-fold increase in steady-state flux and a markedly reduced lag time. This permeation enhancement was further supported by an in vitro lipolysis study, which showed that the nanoemulsion retained 78.4 ± 2.6% of the drug dose in the aqueous micellar phase following simulated intestinal digestion, compared with only 2.8 ± 0.5% for the plain drug suspension indicating that the dual-oil matrix (Vippa oil and Sunflower oil) effectively maintains Artemether in a solubilized, absorbable state throughout digestion rather than allowing it to precipitate as would occur with conventional oral dosage forms.

Collectively, these findings indicate that the optimized Artemether nanoemulsion possesses favorable physicochemical, release, digestion, and ex vivo permeation characteristics that support its potential as an oral delivery platform for this poorly water-soluble antimalarial drug. However, these conclusions are based entirely on in vitro, ex vivo, and accelerated stability data; no in vivo pharmacokinetic or pharmacodynamic evaluation, long-term (room-temperature, real-time) stability study, or pilot-scale/scale-up manufacturability assessment was performed as part of this work. Consequently, the extent to which the observed permeation and digestion enhancements translate into improved in vivo oral bioavailability, and whether the formulation remains stable and reproducible upon scale-up, cannot yet be confirmed. Future studies incorporating in vivo pharmacokinetic evaluation in a suitable animal model, long-term real-time stability testing under ICH-recommended storage conditions, and pilot-scale batch manufacture are recommended to validate the in vitro and ex vivo findings reported here and to support the translational development of this formulation as a viable oral antimalarial delivery system.

## Figures and Tables

**Table 1:** Quantitative saturation solubility of Artemether in various solvents and formulation excipients at 25 °C (*n* = 3).

Solvent/Excipient	Solubility (mg/mL)
Distilled Water	0.02 ± 0.01
Phosphate Buffer (pH 6.8)	0.15 ± 0.03
Methanol	85.40 ± 2.35
Ethanol	72.65 ± 1.82
Chloroform	250.50 ± 5.40
Sunflower Oil	45.25 ± 1.54
Vippa Oil	68.40 ± 2.15
Tween 80	55.30 ± 1.75
Span 80	62.15 ± 1.90

**Table 2:** Selection of independent variables and their levels for BBD.

Independent Variables					
Factor	Name	Units	Low Level (−1)	Medium (0)	High Level (+1)
A	Aqueous phase	%	75	80	85
B	Oil:Surfactant	%	1	1.5	2
C	Sonication time	min	5	7.5	10
Dependent variables (Responses)			Limit		
R1	Particle size (PS)	nm	<500 nm		
R2	Poly Dispersity Index (PDI)		<1		

**Table 3:** Experimental runs and observed responses for the Box–Behnken design.

		Factor 1	Factor 2	Factor 3	Response 1	Response 2
Std	Run	A: aqs. Phase (mL)	B: Oil-Surfactant (%)	C: Sonication Time (min)	PS (nm)	PDI
1	2	75	1	7.5	147.1 ± 2.5	0.276 ± 0.8
2	12	85	1	7.5	149.3 ± 4.2	0.309 ± 1.1
3	15	75	2	7.5	145.3 ± 3.1	0.250 ± 0.9
4	10	85	2	7.5	144.2 ± 1.8	0.314 ± 0.5
5	4	75	1.5	5	144.5 ± 2.2	0.247 ± 1.2
6	5	85	1.5	5	143.7 ± 1.8	0.297 ± 0.7
7	9	75	1.5	10	139.5 ± 1.0	0.277 ± 0.4
8	14	85	1.5	10	154.9 ± 3.8	0.311 ± 0.9
9	6	80	1	5	144.1 ± 1.9	0.321 ± 0.4
10	1	80	2	5	135.8 ± 1.1	0.324 ± 0.8
11	16	80	1	10	139.6 ± 1.3	0.256 ± 0.3
12	8	80	2	10	145.6 ± 2.1	0.304 ± 0.6
13	7	80	1.5	7.5	142.8 ± 1.4	0.298 ± 1.1
14	11	80	1.5	7.5	142.8 ± 2.4	0.298 ± 1.5
15	17	80	1.5	7.5	142.8 ± 1.8	0.298 ± 0.6
16	3	80	1.5	7.5	142.8 ± 1.0	0.298 ± 0.4
17	13	80	1.5	7.5	142.8 ± 1.3	0.298 ± 0.3

Each formulation was measured in triplicate (*n* = 3).

**Table 4:** Physicochemical properties.

Parameter	Measured Value
pH	6.4 ± 0.2
Viscosity (cP)	2.84 ± 0.15
Refractive Index	1.338 ± 0.002
Conductivity (µS/cm)	215 ± 12

**Table 5:** Drug content (F1–F17).

Formulation Type	% Drug Content
F1	88.50 ± 1.7
F2	86.12 ± 0.9
F3	90.75 ± 1.5
F4	89.87 ± 1.8
F5	94.25 ± 0.1
F6	98.44 ± 0.2
F7	95.86 ± 1.4
F8	96.46 ± 1.8
F9	88.06 ± 2.0
F10	89.48 ± 2.1
F11	88.70 ± 1.6
F12	90.60 ± 0.7
F13	88.54 ± 2.1
F14	88.54 ± 2.1
F15	88.54 ± 2.1
F16	88.54 ± 2.1
F17	88.54 ± 2.1

**Table 6:** Percentage of ART dose distributed across digestion phases at 30 min of simulated intestinal lipolysis (mean ± SD, *n* = 3).

Formulation	% in Aqueous Micellar Phase	% in Oil Phase	% Precipitated
Plain ART Suspension	2.8 ± 0.5	—	97.2 ± 0.5
Optimized ART-NE	78.4 ± 2.6	16.2 ± 1.4	5.4 ± 0.9

**Table 7:** In vitro drug release of ART-NEs.

Time (h)	F1	F2	F3	F4	F5	F6	F7	F8	F9	F10	F11	F12	F13	F14	F15	F16	F17
0	0	0	0	0	0	0	0	0	0	0	0	0	0	0	0	0	0
0.5	3.23 ± 0.40	3.19 ± 0.18	3.09 ± 0.16	3.31 ± 0.18	3.27 ± 0.23	3.81 ± 0.72	3.68 ± 0.88	3.46 ± 0.32	4.37 ± 0.12	3.46 ± 0.51	3.45 ± 0.26	3.22 ± 0.15	3.08 ± 0.16	4.12 ± 0.40	3.19 ± 0.17	3.02 ± 0.21	3.40 ± 0.51
1	8.54 ± 0.60	9.90 ± 0.21	10.01 ± 0.72	10.17 ± 0.36	9.49 ± 0.31	10.52 ± 0.22	11.91 ± 0.72	10.37 ± 0.33	12.10 ± 0.21	11.96 ± 0.65	10.45 ± 0.29	12.27 ± 0.16	10.47 ± 0.22	11.60 ± 0.18	9.91 ± 0.24	10.40 ± 0.47	12.24 ± 0.40
2	18.57 ± 0.20	15.68 ± 0.19	18.14 ± 0.17	16.88 ± 0.22	21.42 ± 0.76	22.42 ± 0.22	21.42 ± 0.99	19.92 ± 0.26	22.00 ± 0.15	21.42 ± 0.78	18.12 ± 0.76	21.42 ± 0.25	20.24 ± 0.26	22.47 ± 0.19	16.65 ± 0.18	19.81 ± 0.26	21.49 ± 0.61
3	22.96 ± 0.11	28.11 ± 0.53	28.94 ± 0.35	22.96 ± 0.63	36.50 ± 0.11	36.51 ± 0.43	30.06 ± 0.12	31.45 ± 0.13	30.24 ± 0.32	29.92 ± 0.54	29.44 ± 0.28	28.44 ± 0.43	29.44 ± 0.82	32.70 ± 0.35	25.53 ± 0.57	29.19 ± 0.51	29.31 ± 0.26
4	31.24 ± 0.14	34.38 ± 0.23	35.42 ± 0.28	31.24 ± 0.26	45.97 ± 0.44	41.87 ± 0.17	39.42 ± 0.23	38.91 ± 0.32	39.09 ± 0.54	39.09 ± 0.89	36.32 ± 0.10	38.77 ± 0.65	40.06 ± 0.86	40.16 ± 0.31	32.60 ± 0.28	38.63 ± 0.59	39.26 ± 0.42
5	37.36 ± 0.17	41.70 ± 0.75	43.98 ± 0.36	45.76 ± 0.51	50.65 ± 0.89	58.79 ± 0.54	44.62 ± 0.54	43.66 ± 0.46	43.34 ± 0.72	43.52 ± 0.76	40.39 ± 0.82	42.84 ± 0.69	42.37 ± 0.36	49.84 ± 0.67	43.89 ± 0.64	42.63 ± 0.36	43.93 ± 0.61
6	45.76 ± 0.43	59.73 ± 0.13	56.75 ± 0.14	55.66 ± 0.88	57.79 ± 0.22	66.18 ± 0.65	53.31 ± 0.34	51.64 ± 0.35	54.79 ± 0.54	52.97 ± 0.35	52.52 ± 0.83	54.00 ± 0.38	55.08 ± 0.20	59.50 ± 0.59	57.45 ± 0.45	55.34 ± 0.16	53.78 ± 0.37
7	52.02 ± 0.18	66.60 ± 0.64	62.49 ± 0.10	67.04 ± 0.21	63.31 ± 0.11	72.14 ± 0.12	61.02 ± 0.22	60.04 ± 0.49	58.86 ± 0.94	60.84 ± 0.41	56.44 ± 0.21	60.19 ± 0.35	60.51 ± 0.82	64.08 ± 0.53	66.86 ± 0.43	61.27 ± 0.46	60.55 ± 0.34
8	65.15 ± 0.73	71.64 ± 0.32	69.04 ± 0.86	74.89 ± 0.34	71.19 ± 0.54	79.96 ± 0.42	70.00 ± 0.21	71.17 ± 0.89	68.84 ± 0.16	69.02 ± 0.44	66.24 ± 0.24	71.49 ± 0.51	67.89 ± 0.63	73.40 ± 0.25	72.87 ± 0.32	68.51 ± 0.69	70.53 ± 0.37
9	72.39 ± 0.64	79.84 ± 0.63	78.24 ± 0.34	79.37 ± 0.22	78.73 ± 0.44	82.24 ± 0.33	77.84 ± 0.93	78.19 ± 0.32	78.69 ± 0.44	78.51 ± 0.25	76.57 ± 0.31	76.39 ± 0.95	77.04 ± 0.34	80.02 ± 0.37	79.34 ± 0.40	77.39 ± 0.33	77.18 ± 0.95
10	84.94 ± 0.27	83.57 ± 0.66	85.88 ± 0.76	87.10 ± 0.41	83.19 ± 0.33	88.62 ± 0.36	84.74 ± 0.11	86.72 ± 0.66	85.71 ± 0.51	85.89 ± 0.54	83.26 ± 0.62	86.04 ± 0.94	83.59 ± 0.63	86.78 ± 0.44	85.37 ± 0.55	84.48 ± 0.71	84.97 ± 0.52
11	89.36 ± 0.65	88.74 ± 0.32	90.38 ± 0.35	92.49 ± 0.44	88.90 ± 0.22	91.37 ± 0.86	90.80 ± 0.42	91.62 ± 0.31	91.44 ± 0.85	92.11 ± 0.93	87.84 ± 0.71	90.00 ± 0.16	90.80 ± 0.76	91.46 ± 0.82	90.76 ± 0.41	90.75 ± 0.60	90.54 ± 0.23
12	94.76 ± 0.83	93.80 ± 0.77	94.25 ± 0.31	95.73 ± 0.65	93.58 ± 0.63	97.90 ± 0.45	96.72 ± 0.99	95.64 ± 0.78	96.69 ± 0.66	94.14 ± 0.71	95.84 ± 0.33	94.23 ± 0.26	97.02 ± 0.26	96.79 ± 0.57	94.80 ± 0.74	96.06 ± 0.27	95.78 ± 0.62

**Table 8:** Ex vivo drug release of optimized formulation.

Time (h)	% Cumulative Drug Release (% CDR)
0.5	3.40 ± 0.18
1	12.17 ± 0.72
2	25.00 ± 0.22
3	33.29 ± 0.63
4	41.48 ± 0.78
5	46.76 ± 0.32
6	58.08 ± 0.39
7	61.20 ± 0.71
8	63.68 ± 0.67
9	70.15 ± 0.22
10	84.87 ± 0.87
11	89.09 ± 0.51
12	92.21 ± 0.97

**Table 9:** Ex vivo permeation parameters of plain ART suspension versus optimized ART nanoemulsion across excised goat intestinal mucosa (mean ± SD, n = 3).

Parameter	Plain ART Suspension (Control)	Optimized ART Nanoemulsion
Steady-State Flux (Jss)	145.20 ± 6.14 µg/cm^2^/h	586.45 ± 12.38 µg/cm^2^/h
Apparent Permeability (Papp)	(2.52 ± 0.11) × 10^−5^ cm/s	(1.02 ± 0.04) × 10^−4^ cm/s
Lag Time	1.85 ± 0.14 h	0.42 ± 0.06 h
Enhancement Ratio	1.00	4.04 ± 0.18
Cumulative Permeation (12 h)	24.35 ± 1.42%	92.21 ± 0.97%

**Table 10:** Zero-order, first-order, Higuchi, and Korsmeyer–Peppas kinetic plots of ART-NEs (F1–F17).

Formulation	Zero-Order R2	First-Order R2	Higuchi R2	Korsmeyer–Peppas	
				R2	N
F1	0.995	0.781	0.919	0.991	0.30
F2	0.983	0.745	0.947	0.987	0.28
F3	0.992	0.735	0.953	0.996	0.39
F4	0.987	0.760	0.937	0.987	0.36
F5	0.974	0.689	0.973	0.974	0.13
F6	0.963	0.684	0.970	0.978	0.25
F7	0.994	0.566	0.958	0.921	0.20
F8	0.993	0.708	0.953	0.998	0.19
F9	0.994	0.731	0.955	0.996	0.31
F10	0.992	0.720	0.954	0.991	0.27
F11	0.995	0.727	0.946	0.987	0.15
F12	0.992	0.714	0.955	0.982	0.18
F13	0.994	0.709	0.955	0.988	0.34
F14	0.993	0.715	0.952	0.990	0.26
F15	0.989	0.742	0.941	0.993	0.33
F16	0.995	0.720	0.949	0.985	0.19
F17	0.971	0.686	0.972	0.976	0.21

**Table 11:** ANOVA for the quadratic model of particle size.

Source	Sum of Squares	df	Mean Square	F-Value	*p*-Value	
Model	253.51	9	28.17	7.63	0.0069	significant
A—Aqueous Phase	30.81	1	30.81	8.35	0.0233	
B—Oil:Surfactant	10.58	1	10.58	2.87	0.1342	
C—Sonication Time	16.53	1	16.53	4.48	0.0721	
AB	2.72	1	2.72	0.7379	0.4188	
AC	65.61	1	65.61	17.78	0.0040	
BC	51.12	1	51.12	13.86	0.0074	
A^2^	73.39	1	73.39	19.89	0.0029	
B^2^	0.1684	1	0.1684	0.0456	0.8369	
C^2^	4.42	1	4.42	1.20	0.3098	
Residual	25.83	7	3.69			
Lack of Fit	25.53	3	8.51	113.46	0.0003	significant
Pure Error	0.3000	4	0.0750			
Cor Total	279.34	16				

**Table 12:** Fit statistics of particle size.

Std. Dev.	1.92	R^2^	0.9075
Mean	143.89	Adjusted R^2^	0.7887
C.V. %	1.33	Predicted R^2^	−0.4639
		Adeq Precision	12.2096

**Table 13:** ANOVA for the linear model of PDI.

Source	Sum of Squares	df	Mean Square	F-Value	*p*-Value	
Model	0.0044	3	0.0015	3.73	0.0392	significant
A—Aqueous Phase	0.0041	1	0.0041	10.37	0.0067	
B—Oil:Surfactant	0.0001	1	0.0001	0.2847	0.6026	
C—Sonication Time	0.0002	1	0.0002	0.5318	0.4788	
Residual	0.0051	13	0.0004			
Lack of Fit	0.0050	9	0.0006	15.28	0.0093	significant
Pure Error	0.0001	4	0.0000			
Cor Total	0.0096	16				

**Table 14:** Fit Statistics of PDI.

Std. Dev.	0.0199	R^2^	0.4624
Mean	0.2946	Adjusted R^2^	0.3383
C.V. %	6.75	Predicted R^2^	−0.0607
		Adeq Precision	5.7563

Note: For PDI, the model showed a low coefficient of determination (R^2^ = 0.4624), with an adjusted R^2^ of 0.3383 and a negative predicted R^2^ (−0.0607), indicating that the quadratic model explains only a small portion of the variability in PDI and has limited predictive ability. This is expected given the narrow range of PDI observed across the formulations (0.247–0.324), which restricts the model’s ability to discriminate between runs. Nevertheless, the adequate precision value of 5.76 (>4) confirms an adequate signal-to-noise ratio, indicating that the model is still usable for navigating the design space. Importantly, all PDI values remained well below 1.0, confirming a narrow and homogeneous droplet size distribution across all formulations.

**Table 15:** Regression coefficients in terms of coded factors for particle size (Y_1_) and polydispersity index (Y_2_).

Factor	Coefficient Estimation for Y_1_ (Particle Size)	Coefficient Estimation for Y_2_ (Polydispersity Index)
Intercept	141.73	0.2950
A: Aqueous Phase	1.96	0.0226
B: Oil: Surfactant	−1.15	0.0038
C: Sonication time	1.44	−0.0059
AB	−0.8250	0.0077
AC	4.05	−0.0040
BC	3.57	0.0112
A^2^	4.56	−0.0130
B^2^	0.1875	0.0052
C^2^	−0.6375	0.0010

**Table 16:** Constraints applied for numerical optimization.

Name	Goal	Lower Limit	Upper limit	Lower Weight	Upper Weight	Importance
A: Aqueous Phase (mL)	Minimize	75	85	**1**	1	3
B: Oil:Surfactant (mL)	Is in range	1	2	**1**	1	3
C: Sonication Time (min)	Maximize	5	10	**1**	1	3
PS (nm)	Minimize	135.8	154.9	**1**	1	3
PDI (cm^2^/vs)	Minimize	0.247	0.324	**1**	1	3

**Table 17:** Optimized solutions selected after assessment of the design space.

N.	Aqueous Phase	Oil: Surfactant	Sonication Time	PS	PDI	Desirability
1	75.000	1.000	10.000	138.013	0.260	0.925
2	75.000	1.013	10.000	137.994	0.258	0.934
3	75.000	1.036	10.000	139.900	0.257	0.908
4	75.051	1.000	9.990	142.133	0.264	0.781

**Table 18:** Predicted and error values.

S. No	Response	Predicted Value	Obtained Value	% Error
1	Particle Size (PS)	138.013	139.5	0.913
2	Polydispersity Index (PDI)	0.260	0.256	1.54

**Table 19:** Stability study results of nanoemulsion formulation over 9 months.

Time Point	Particle Size (nm)	PDI	Zeta Potential (mV)	Drug Content (%)
Initial (Day 0)	139.6 ± 1.3	0.256 ± 0.03	−30.08 ± 1.1	98.44 ± 0.2
3 months	148 ± 3	0.30 ± 0.02	−27.5 ± 1.2	96.5 ± 0.8
6 months	155 ± 5	0.33 ± 0.03	−25.0 ± 1.5	95.0 ± 1.0
9 months	165 ± 7	0.38 ± 0.04	−22.0 ± 2.0	92.5 ± 1.5

Storage condition assumed: accelerated stability at 40 ± 2 °C/75 ± 5% RH.

**Table 20:** Quality Target Product Profile (QTPP) for the ART nanoemulsion.

QTPP Element	Target
Dosage form	Oil-in-water (O/W) nanoemulsion for oral administration
Route of administration	Oral
Droplet size	<200 nm (nanometric range for enhanced dissolution/permeation)
Physical stability	Homogeneous, non-separating system over the intended shelf life
Drug loading	High and consistent drug content/entrapment across batches
Release profile	Sustained, controlled release over 12 h
Permeation	Enhanced intestinal permeation relative to conventional oral ART formulations

**Table 21:** Qualitative risk assessment linking CMAs/CPPs to CQAs.

Material/Process Attribute	Particle Size	PDI	Zeta Potential	Drug Content/EE%	Risk Level
Oil phase ratio (CMA)	Medium	Low	Low	High	Medium
Surfactant ratio (CMA)	High	Medium	High	Medium	High
Aqueous phase volume (CPP)	High	High	Low	Low	High
Oil:surfactant ratio (CPP)	High	Medium	Low	Low	High
Sonication time (CPP)	High	High	Low	Low	High

## Data Availability

The original contributions presented in this study are included in the article. Further inquiries can be directed to the corresponding author.

## References

[1] Tadros T.F. (2008). Colloids in Cosmetics and Personal Care.

[2] Chhabra G., Chuttani K., Mishra A.K., Pathak K. (2011). Design and development of nanoemulsion drug delivery system of amlodipine besilate for improvement of oral bioavailability. Drug Dev. Ind. Pharm..

[3] Singh K.K., Vingkar S.K. (2008). Formulation, antimalarial activity and biodistribution of oral lipid nanoemulsion of primaquine. Int. J. Pharm..

[4] Morales D., Gutiérrez J.M., García-Celma M.J., Solans Y.C. (2003). A study of the relation between bicontinuous microemulsions and oil/water nano-emulsion formation. Langmuir.

[5] Sharma N., Bansal M., Visht S., Sharma P.K., Kulkarni G.T. (2010). Nanoemulsion: A new concept of delivery system. Chron. Young Sci..

[6] Garg A., Tomar D.S., Bhalala K., Wahajuddin M. (2020). Development and Investigation of Artemether Loaded Binary Solid Lipid Nanoparticles: Physicochemical Characterization and In-Situ Single-Pass Intestinal Permeability. J. Drug Deliv. Sci. Technol..

[7] Laxmi M., Bhardwaj A., Mehta S., Mehta A. (2015). Development and Characterization of Nanoemulsion as Carrier for the Enhancement of Bioavailability of Artemether. Artif. Cells Nanomed. Biotechnol..

[8] Dwivedi P., Khatik R., Chaturvedi P., Khandelwal K., Taneja I., Raju K.S.R., Dwivedi H., Singh S.K., Gupta P.K., Shukla P. (2015). Arteether Nanoemulsion for Enhanced Efficacy against Plasmodium yoelii nigeriensis Malaria: An Approach by Enhanced Bioavailability. Colloids Surf. B Biointerfaces.

[9] Dong L., Ding J., Zhu L., Liu Y., Gao X., Zhou W. (2023). Copper carbonate nanoparticles as an effective biomineralized carrier to load macromolecular drugs for multimodal therapy. Chin. Chem. Lett..

[10] Wang H., Tang C., Zhang H., Guo L., Zou C., Zhang S., Liu J., Huang Q., Liu Y., Zhou W. (2025). A biocompatible tea polyphenol nanoplatform for efficient cytosolic delivery of protein therapeutics. J. Control. Release.

[11] Sai M.K., Nagashubha B., Pallavi R.G., Roopeswari Y. (2026). Formulation and Comprehensive Characterization of a Stable Wintergreen Oil Nano Emulsion with Enhanced Antioxidant Properties for Topical Delivery. J. Pharm. Innov..

[12] Chimanuka B., Gabriels M., Detaevernier M.R., Plaizier-Vercammen J.A. (2002). Preparation of beta-artemether liposomes, their HPLC-UV evaluation and relevance for clearing recrudescent parasitaemia in *Plasmodium chabaudi* malaria-infected mice. J. Pharm. Biomed. Anal..

[13] Nikolic I., Mitsou E., Damjanovic A., Papadimitriou V., Stankovic J.A., Stanojevic B., Xenakis A., Savic S. (2020). Curcumin-loaded low-energy nanoemulsions: Linking EPR spectroscopy-analysed microstructure and antioxidant potential with in vitro evaluated biological activity. J. Mol. Liq..

[14] Kuo F., Subramanian B., Kotyla T., Wilson T.A., Yoganathan S., Nicolosi R.J. (2008). Nanoemulsion of an anti-oxidant synergy formulation containing gamma tocopherol has enhanced bioavailability and antiinflammatory properties. Int. J. Pharm..

[15] Hashtjin A.M., Abbasi S. (2015). Optimization of ultrasonic emulsification conditions for the production of orange peel essential oil nanoemulsions. J. Food Sci. Technol..

[16] Jadhav A.J., Holkar C.R., Karekar S.E., Pinjari D.V., Pandit A.B. (2015). Ultrasoun assisted manufacturing of paraffin wax nanoemulsions: Process Optimization. Ultrason. Sonochem..

[17] Tan S.F., Masoumi G.R.F., Karjiban R.A., Stanslas J., Kirby B.P., Basri M., Bin B.H. (2016). Ultrasonic emulsification of parenteral valproic acid-loaded nanoemulsion with response surface methodology and evaluation of its stability. Ultrason. Sonochem..

[18] Piriyaprasarth S., Sriamornsak P., Chansiri G., Promboot W., Imerb U., Sumpoung D. (2012). Effect of coconut oil and surfactants on stability of nanoemulsions. Adv. Mater. Res..

[19] Carpenter J., Saharan V.K. (2017). Ultrasonic assisted formation and stability of mustard oil in water nanoemulsion: Effect of process parameters and their optimization. Ultrason. Sonochem..

